# On-Resin Synthesis and Diversification of Macrocyclic Disulfide Bridge Peptidomimetics via Bifunctional 5‑Iodo-1,4-Triazoles

**DOI:** 10.1021/acs.joc.6c00195

**Published:** 2026-05-27

**Authors:** Michael A. Malone, Oscar A. Shepperson, Jacob E. Simms, Andrew J. Reid, Andrew G. Jamieson

**Affiliations:** School of Chemistry, Advanced Research Centre, 3526 University of Glasgow, 11 Chapel Lane, Glasgow G11 6EW, U.K.

## Abstract

Cyclic peptides represent an important class of therapeutic molecules, however, often require late-stage functionalization to address suboptimal pharmacokinetic properties. Solid phase bifunctional cyclization strategies that enable both macrocyclization and diversification within a single scaffold remain limited. Herein, we report a general on-resin platform that exploits the 5-iodo-1,4-triazole as a bifunctional macrocyclic disulfide bridge mimetic, enabling modular late-stage modification of cyclic peptides. The methodology was demonstrated across a diverse panel of peptides spanning 6–12 residue macrocycles, incorporating varied amino acid side chains, N-terminal protecting groups, and peptide topologies. Formation of the 5-iodo-1,4-triazole was compatible with functionally diverse sequences under Fmoc-based solid-phase peptide synthesis conditions, highlighting the robustness of the cyclization strategy. Optimization of on-resin Suzuki–Miyaura cross-coupling significantly reduced reaction times while maintaining high conversions, enabling efficient aryl diversification of the triazole linkage. To expand structural scope, a complementary on-resin Sonogashira cross-coupling protocol was developed, allowing direct installation of alkyl linkers, functional handles, and biologically relevant motifs that are inaccessible through aryl-based cross-coupling alone. Together, these orthogonal palladium-catalyzed transformations establish a unified, operationally simple platform for the synthesis and late-stage diversification of cyclic peptidomimetics, providing rapid access to structurally diverse macrocyclic peptides relevant to chemical biology and medicinal chemistry.

## Graphical abstract

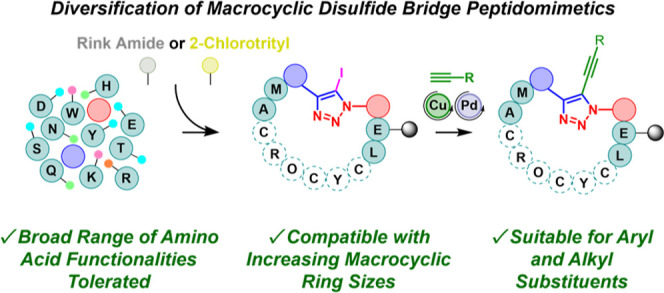

## Introduction

Cyclic peptides have emerged as a powerful class of therapeutic agents, combining the high target specificity and favorable toxicity profiles of linear peptides with enhanced structural and functional properties.^1,2^ Advances in high-throughput discovery platforms, including mRNA display and DNA-encoded libraries (DELs), have greatly expanded access to diverse peptide scaffolds and enabled the routine identification of high-value cyclic peptide hits.^3,4^ Crucially, macrocyclization is now recognized as a central strategy to overcome the intrinsic limitations of linear peptides, including poor cell permeability, limited metabolic stability, and low oral bioavailability.^5^ By conformationally constraining the peptide backbone, cyclization can improve proteolytic resistance and promote productive target engagement. As a result, a wide range of synthetic cyclization chemistries, such as thioether formation, lactamization, and 1,2,3-triazole linkage, have become key peptidomimetic modalities in modern peptide drug design.^6,7^

Despite the success of macrocyclization, peptide biopolymers frequently retain pharmacokinetic liabilities, requiring functionalization at multiple positions to achieve suitable in vivo properties.^8^ Extensive structural modifications can be detrimental to the intrinsic biological activity of peptide scaffolds. This challenge highlights the need for bifunctional peptidomimetic cyclization strategies that enable efficient functionalization while minimizing disruption to bioactive conformations and binding motifs.^9^ Although notable advances have been reported, macrocyclization strategies that exploit bifunctional moieties remain constrained by limited sequence adaptability, restricted macrocycle size, nonorthogonal reactivity, and frequent reliance on solution-phase chemistry.^10,11^

Among established peptidomimetic linkages, 1,2,3-triazoles have emerged as privileged scaffolds in chemical biology and drug discovery, finding broad application in foldamer architectures, antimicrobial peptides, and peptide-drug conjugates.^12–15^ Both copper- and ruthenium-catalyzed azide–alkyne cycloaddition reactions (CuAAC and RuAAC) have been widely employed to generate triazole linkages that effectively mimic amide and disulfide bridge functionality. The extensive study and broad application of these reactions raise the question of whether such motifs may be further exploited as bifunctional peptidomimetic scaffolds. Accordingly, we sought to extend this established cyclization strategy to enable greater functional versatility in peptide systems.

Copper mediated functionalization of the 5-position of 1,4-triazoles has previously been reported for small molecules, including the single-step installation of halogen, aryl, vinyl, and carbonyl substituents.^16–20^ Building on these advances, we recently translated this chemistry through the development of an on-resin approach to 5-iodo-1,4-triazole peptidomimetics, demonstrated using the cyclic peptide atosiban (**1**). Introduction of a reactive iodine handle at the 5-position enabled Suzuki–Miyaura cross-coupling reactions for late-stage diversification, while retaining the triazole as an effective disulfide-bridge mimetic (Figure 1A).^21^

**Figure 1:**
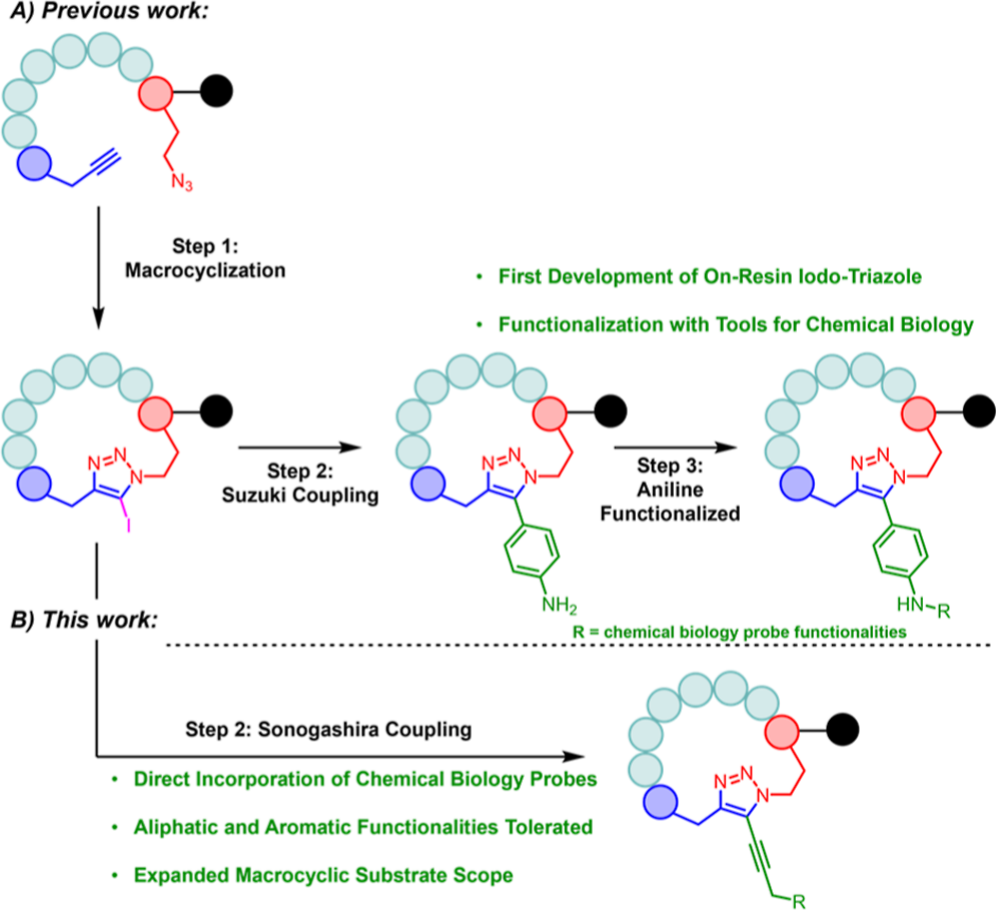
(A) Our previous on-resin functionalization of the 5-iodo-triazole with chemical biological probes via a Pd-catalyzed Suzuki–Miyaura cross coupling followed by an amide coupling. (B) An optimized direct on-resin functionalization of the 5-iodo-triazole with alkynes via a Cu/Pd-catalyzed Sonogashira cross coupling.

Herein, we report the extension of this on-resin methodology to a diverse set of cyclic peptides encompassing a range of ring sizes, thereby demonstrating the generality of the bifunctional trisubstituted triazole scaffold.^21^ Application of the 5-iodo-1,4-triazole-forming chemistry in the presence of functionally diverse amino acid residues further underscores the robustness of the approach. In addition, we have optimized the on-resin Suzuki–Miyaura cross-coupling conditions, substantially reducing reaction times and improving operational efficiency for late-stage peptide functionalization. Building on the inherent reactivity of the 5-iodo-1,4-triazole, we further introduce a complementary on-resin Sonogashira functionalization protocol (Figure 1B), enabling direct installation of alkyne-containing motifs without the need for intermediate handles.^22,23^ Together, these advances establish a unified, on-resin, bifunctional triazole-based macrocyclization and diversification platform that enables modular late-stage functionalization of cyclic peptides, representing a general and operationally simple strategy for peptide modification.

## Results and Discussion

### Macrocyclization Compatibility for On-Resin 5-Iodo-1,4-Triazole Formation

To demonstrate the versatility of our on-resin 5-iodo-1,4-triazole chemistry, we selected four structurally distinct peptide targets: an oxytocin mimetic (**2**), a sunflower trypsin inhibitor-1 analogue (SFTI) (**3**), a cyclic RGD octapeptide (**4**) and somatostatin (**5**).^24–27^ Together, these peptides span a wide range of ring sizes, topologies, and biological functions, providing a rigorous testbed for evaluating the scope of the methodology. These four sequences feature disulfide macrocyclic rings spanning 6 to 12 amino acid residues, extending the scope beyond the previously demonstrated 22-atom system to include 28-, 34-, and 40-atom macrocycles (Figure 2). Furthermore, a diverse range of amino acids were incorporated across the four peptides. Cysteine residues were deliberately omitted, as they were replaced with non-natural amino acids to install the triazole motifs. Methionine was omitted due to complications associated with oxidative side reactions. The remaining 18 proteinogenic amino acids were incorporated throughout the peptide chains. Additionally, C-terminal acids and amides were investigated, alongside the standard suite of side chain protecting groups commonly used in Fmoc/^*t*^Bu solid-phase peptide synthesis (Fmoc-SPPS). This deliberate diversification allowed for the strict interrogation of the compatibility of this chemistry with ring size, side chain functionalities and protecting groups.

**Figure 2:**
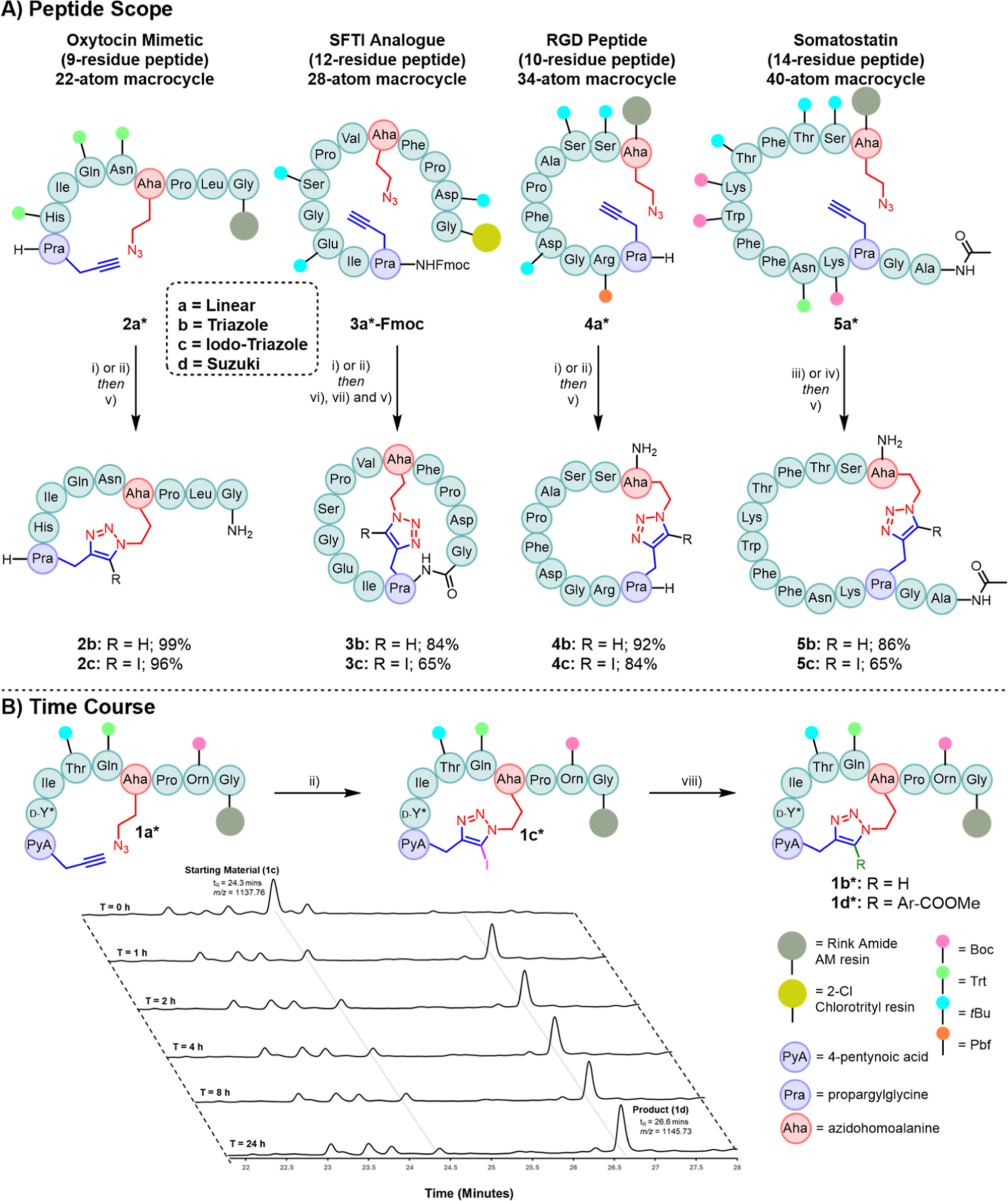
(A) Scope of peptides included herein with variable macrocyclic ring size of 6–12 residues. (B) Time course study for the formation of Suzuki functionalized on-resin peptide 1d* by RP-HPLC. Reagents and conditions: (i) CuI, NaAsc, DIPEA, TBTA and 2,6-lutidine in DMF/TFE (1:1, *v/v*), 55 °C, 10 min; (ii) CuI, DIPEA and NBS in DMF, r.t., 18 h; (iii) CuI, NaAsc, DIPEA, TBTA and 2,6-lutidine in DMF/DMSO (1:1, *v/v*), 55 °C, 2 h; (iv) CuI, DIPEA and NBS in DMF/DMSO (1:1, *v/v*), r.t., 18 h; (v) TFA/TIPS:H_2_O (95:2.5:2.5, *v/v/v*), r.t., 2 h; (vi) Piperidine and formic acid in DMF (75:20:5, *v/v/v*), r.t., 2 × 10 min; (vii) CH_2_Cl_2_:TFA (99:1, *v/v*), r.t., 2 h, followed by, HATU and DIPEA in DMF, r.t., 3 h; (viii) 4-methoxycarbonylphenylboronic acid pinacol ester, Pd­(PPh_3_)_4_, K_3_PO_4_.3H_2_O in DMF/H_2_O (95:5, *v/v*), Argon, 60 °C, 1 h. * denotes on-resin peptide.

All linear peptides (1a*–5a*) were synthesized via automated Fmoc-SPPS. Peptide 1a* was synthesized as previously reported (where * denotes the on-resin peptide).^21^ Azide and alkyne functionalities were incorporated into 2a*–5a* via the inclusion of azidohomoalanine (Aha) and either 4-pentynoic acid (PyA) or propargylglycine (Pra) respectively. Previous work surrounding the 1,4-triazole disulfide bridge mimetic demonstrates that the optimal bridge conformation is achieved with Aha located proximal to the C-terminus, hence this was maintained for all analogues synthesized herein.^28^

To assess the compatibility of our chemistry with commonly employed N-terminal peptide functionalities, a range of protecting groups was examined. The linear peptides **2a–5a** were synthesized with the following N-terminal protection; Fmoc for the oxytocin mimetic (**2**) and sunflower trypsin inhibitor (**3**), Boc for the RGD octapeptide (**4**), and acetyl-capped somatostatin (**5**).

Peptides 2a*–5a* were initially subjected to standard on-resin CuAAC conditions to obtain 1,4-triazole cyclized analogues 2b*–5b*. As formation of the 1,4-triazole does not result in an observable mass change, product formation was monitored by a shift in retention time on RP-HPLC. The 1,4-triazole cyclization reactions enabled evaluation of the propensity of each peptide to undergo efficient macrocyclization. Furthermore, such reactions provided a baseline for monitoring conversion when applying our modified CuAAC conditions.^21,26^ Pleasingly, 2a*–4a* efficiently underwent the 1,4-triazole cyclization to 2b*–4b* (84–99%) (Figure 2A). However, linear peptide 5a* required modified CuAAC conditions to effect macrocyclization, including extending the reaction time to 2 h and employing a DMF/DMSO (1:1, *v/v)* solvent mixture to promote solvation. Under these conditions a satisfactory conversion to 1,4-triazole 5b* (86%) was achieved. It should be noted that conversion herein and throughout is reporting the consumption of starting material to desired final product and not crude product purity.

Following the successful CuAAC macrocyclization to provide 1,4-triazole analogues 2b*–5b*, we next evaluated the robustness of the on-resin 5-iodo-1,4-triazole-forming protocol across these model peptidyl substrates. Accordingly, peptides 2a*–5a* were subjected to the previously reported standard conditions.^21^ Gratifyingly, 5-iodo-1,4-triazoles 2c*–4c* were formed efficiently, with conversions of 96%, 65%, and 84%, respectively, demonstrating good compatibility with the established cyclization methodology. In contrast, and consistent with observations from the CuAAC macrocyclisation, substrate **5a** required modified conditions. Under the previously optimized protocol, 5a* remained largely unreacted. To address this, a DMF/DMSO (1:1, *v/v*) solvent system at 55 °C was employed, resulting in 56% conversion to 5c*. While reduced conversion for this substrate was anticipated, this result is nonetheless significant, as it highlights a practical strategy to overcome diminished reactivity in longer and/or more hydrophobic macrocycles. Consequently, the optimized conditions for formation of 5c* provide a viable alternative for accessing 5-iodo-1,4-triazole mimetics in sequences enriched in hydrophobic residues or featuring larger macrocyclic architectures.

Following the successful synthesis of each of the desired macrocyclic peptides 2c*–5c*, they were liberated from the resin and purified by RP-HPLC to yield **2a–5c** in high yields and excellent purity. Characterization of each of the peptidyl products **2a–5c** was affirmed by LCMS (Table S1).

### Optimization and Sequence Scope of On-Resin Suzuki–Miyaura Cross-Coupling

Building on our previously reported on-resin Suzuki–Miyaura functionalization of 5-iodo-1,4-triazole peptidomimetics, which achieved sufficient conversion after overnight reaction, we sought to optimize the reaction time to render the chemistry more amenable to on-resin peptide synthesis.^21^ To optimize the overall reaction length, a time-course study was performed using the 5-iodo-1,4-triazole atosiban peptidomimetic 1c*. 4-Methoxycarbonylphenylboronic acid pinacol ester was employed as a model substrate to investigate the time required for complete reaction. Pleasingly, quantitative conversion of the starting material to the desired product (1d*) was observed after only 1 h (Figure 2B).

The time-optimized Suzuki–Miyaura cross coupling procedure was subsequently applied to peptides 2c*–5c*, allowing assessment of the tolerance of various side chains and peptide environments to this protocol. Under the optimized conditions, no formation of 2d* was observed. The reaction was repeated, exchanging the N-terminal Fmoc for Boc-protected 2c*; however, no formation of 2d* was once again observed.^29^ Upon closer examination, 2c* was identified as the only peptide investigated containing a histidine residue within its sequence. Coordination of imidazoles to palladium is well documented to cause catalyst poisoning, including in the case of palladium-mediated transformations on peptides containing histidine.^29^ Application of the Suzuki protocol demonstrated high compatibility with the remaining peptides. Modification of the respective 5-iodo-1,4-triazoles (3c*–5c*) to the corresponding Suzuki product (3d*–5d*) reported favorable conversions (65–81%) (Figure S1). Interestingly, peptide 3c* contains a Fmoc protected N-terminus, which demonstrated a high degree of robustness under such conditions, and further supported that the imidazole of histidine was likely responsible for the lack of reaction observed for peptide **2**. The peptides **3d**–**5d** were obtained, with purity >95% (Table S1).

### Development of On-Resin Sonogashira Cross-Coupling for Alkyl Diversification of 5-Iodo-1,4-Triazoles

Functionalization of the 5-iodo-1,4-triazole via Suzuki–Miyaura cross-coupling has provided a route to bifunctional cyclization mimetics. While the methodology is currently best suited to aryl borylates, owing to constraints imposed by β-hydride elimination, it nevertheless offers a robust platform for introducing a wide range of aromatic functionality.^30^ Rather than relying on a borylated substrate and a large planar aromatic aniline spacer for downstream modification, we aimed to expand the structural flexibility of our bifunctional modality. To achieve this, we sought an alternative cross-coupling strategy that would enable direct installation of linear aliphatic linkers. We anticipated that a Sonogashira modification would meet this goal, providing straightforward access to both aromatic and aliphatic substituents, and enabling the direct attachment of chemical biology probes to the triazole mimetic.

Optimization of the Sonogashira cross-coupling was performed on the on-resin 5-iodo-1,4-triazole peptide substrate 1c* using a range of bases (15 equiv) at 70 °C for 4 h (Table 1, **Entries 1–4**). Under these conditions, all bases afforded high conversion of the on-resin 5-iodo peptidyl intermediate 1c* to the desired Sonogashira product 1e*. Given the widespread use of *N*,*N*-diisopropylethylamine (DIPEA) in Fmoc-SPPS, particularly its compatibility with Fmoc deprotection, we aimed to further optimize the reaction while retaining DIPEA as the base of choice. Reduction of the alkyne from 15 equiv. to 5 equiv. was not well tolerated, however 5 equiv of base maintained excellent conversion. Finally, an attempt using 5 equiv of base and 10 equiv of the alkyne demonstrated the optimal conditions, affording the desired product in 94% conversion (Table 1, **Entries 5–7**). Notably, attempts to reduce the temperature and reaction length were unable to uphold the high degree of conversion to product (Table 1, **Entry 8**) (Figures S2–S4).

**Table 1:**
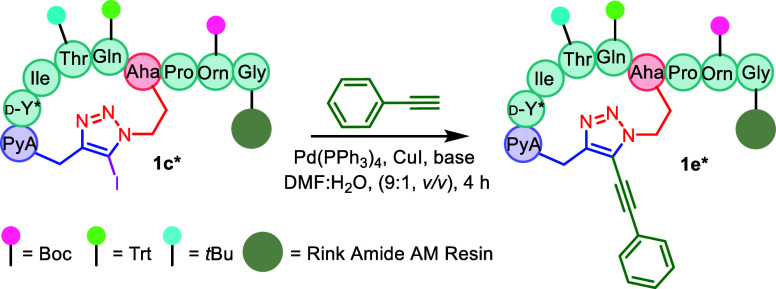
Optimization of On-resin Sonogashira Cross-coupling on 5-Iodo-1,4-Triazole (1c*)

reaction conditions				% area ratio^a^		
entry	alkyne (equiv)	base (equiv)	temp (°C)	1c*	1b*	1e*
**1**	15	DIPEA (15)	70	2	5	97
**2**	15	pyridine (15)	70	0	13	87
**3**	15	Cs_2_CO_3_ (15)	70	1	3	96
**4**	15	K_3_PO_4_.3H_2_O (15)	70	2	2	96
**5**	5	DIPEA (15)	70	5	23	72
**6**	15	DIPEA (5)	70	3	48	49
**7**	10	DIPEA (5)	70	2	4	94
**8**	10	DIPEA (5)	40	5	87	8

a % area determined at 214 nm. *denotes on-resin peptide. d-Y* = d-tyr­(OEt).

### Substrate Scope of On-Resin Sonogashira Cross-Coupling to Functionalize 5-Iodo-1,4-Triazoles

To explore the scope of the newly developed on-resin Sonogashira conditions, we sought to examine a range of functionalized aromatic and aliphatic alkynes. 4-Ethynylaniline was initially chosen as a direct comparison to the recently reported Suzuki–Miyaura chemistry.^21^ Pleasingly, the alkynyl aniline displayed excellent conversion to the desired product, 1f* (Table 2). The previously reported functionalized 5-iodo-1,4-triazole was unable to undergo couplings with substrates bearing acidic moieties. Therefore, under the optimized Sonogashira conditions, 4-ethynyl benzoic acid was examined. Employing the standardized conditions, only 31% conversion to the desired product, 1g*, was obtained. We hypothesized that the excess 4-ethynylbenzoic acid relative to DIPEA was neutralizing the reaction mixture, preventing deprotonation of the copper-coordinated 4-ethynyl benzoic acid. Consequently, the equivalents of base were adjusted (5 equiv. to 15 equiv) to return the system to an overall basic environment. Encouragingly, this adjustment led to a substantial improvement in conversion to 74% (Table 2). In attempts to achieve full consumption of 1c*, the reaction length was extended (7 h). However, this failed to improve product formation (1g*) (Figure S5). Following the successful incorporation of aromatic alkynes, we examined the applicability of flexible alkyl linkers, previously unattainable via Suzuki–Miyaura couplings. Reaction of iodo-1,4-triazole 1c* with 3-butyn-1-ol gave near-complete conversion (97%) to the desired product (Table 2, 1h*). The alkenyl amine substrate, but-3-yn-1-amine (Table 2, 1i*), achieved a 45% conversion. However, subjecting 1c* to two treatments of the reaction conditions, while employing the Fmoc-protected amino-alkyne, afforded 1i* in a 76% conversion. We investigated the incorporation of an alkynyl acid substituent, 4-pentynoic acid, via the optimized Sonogashira cross-coupling conditions. As observed for **1g**, the use of 5 equiv of DIPEA resulted in no detectable formation of 1j*. To increase the effective basicity, the DIPEA loading was raised from 5 to 15 equiv: despite this, no product formation was observed. However, exchanging DIPEA with Cs_2_CO_3_ (mesh 200), a 44% conversion of 1j* was observed (Figure S6).

**Table 2:**
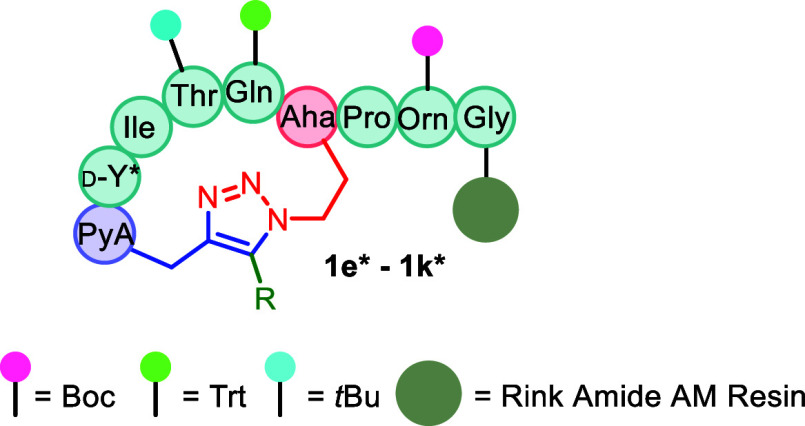

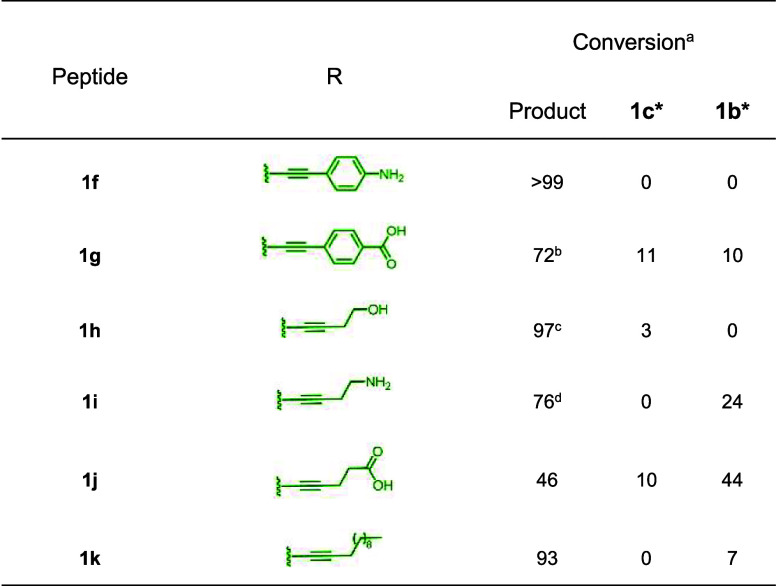
Alkyne Substrate Scope Using Optimized Sonogashira Cross-Coupling Conditions

a % conversion was determined at by observation of analytical RP-HPLC at 214 nm as well as LCMS (Figures S4 and S5).

b 15 equiv of DIPEA were used.

c 15 equiv of Cs_2_CO_3_ were used.

d The peptide was subjected to the reaction conditions to allow two treatments. *denotes the on-resin peptide. d-Y* = d-tyr­(OEt).

The optimized Sonogashira cross-coupling conditions were subsequently applied to the various on-resin peptides 2c*–5c* (Table 1, **Entry 8**). Akin to the on-resin Suzuki–Miyaura coupling, 2c* failed to react under the palladium-catalyzed Sonogashira conditions, presumably due to the inclusion of a histidine residue. Pleasingly, peptides 3c*–5c* displayed high conversions to the desired products under the optimized conditions (67–90% conversion) (Figure S7). Notably, the on-resin, N-terminally Fmoc-protected peptide 3c* showed high compatibility with the reaction conditions, highlighting the suitability of this chemistry for use in Fmoc-based solid-phase peptide synthesis. Following resin cleavage and global deprotection, the desired peptides (**3e** and **4e**) were isolated in moderate to good yields (20–30%). Longer peptides (**5e**) exhibited lower isolated yields (5%), however, this was anticipated due to the increased number of synthetic steps required to assemble the full-length peptides.

Finally, we sought to demonstrate the utility of our chemistry developed herein with the inclusion of a biologically relevant alkyne functionalized moiety. Lipidation is known to enhance the pharmacokinetic properties of peptides; therefore, an alkynyl lipid was investigated.^31^ Sonogashira cross-coupling of the 5-iodo intermediate (1c*) yielded 1k* (93% conversion), highlighting the utility of this chemistry for direct late-stage modification in the synthesis of bifunctional peptidomimetics.

## Conclusion

In summary, we have demonstrated that the 5-iodo-1,4-triazole functions as a general and versatile on-resin macrocyclic disulfide bridge mimetic that enables modular late-stage diversification of cyclic peptides. Extension of our previously reported methodology to a structurally diverse panel of peptides spanning 6–12 residue macrocycles established broad compatibility with ring size, sequence composition, N-terminal protecting groups, and common Fmoc-SPPS conditions. The ability to form 5-iodo-1,4-triazoles in the presence of functionally diverse amino acid side chains highlights the robustness of this approach for peptide macrocyclization.

Optimization of the on-resin Suzuki–Miyaura cross-coupling significantly reduced reaction times while maintaining high conversions across multiple peptide scaffolds, enabling efficient late-stage aryl diversification. Importantly, the development of a complementary on-resin Sonogashira cross-coupling protocol further expanded the structural scope of this platform, allowing direct installation of alkyl linkers, functional handles, and biologically relevant motifs that are inaccessible through aryl-based cross-coupling alone. Together, these orthogonal palladium-catalyzed transformations establish a unified, bifunctional triazole-based cyclization and diversification strategy that is operationally simple and fully compatible with solid-phase peptide synthesis.

This work provides a general chemical platform for the synthesis and late-stage modification of cyclic peptides, enabling rapid access to structurally diverse peptidomimetics relevant to chemical biology, medicinal chemistry, and translational drug discovery, and is expected to facilitate the systematic exploration of structure–function relationships in macrocyclic peptide systems.

## Supplementary Material



## Data Availability

The data underlying this work is available in the published article and its Supporting Information.

## Notes

Detailed experimental procedures, NMR spectra, LCMS and HPLC data, and figures related to the synthesis and purification of for all the compounds synthesized (PDF)
