# Supplementary material for: On-Resin Synthesis and Diversification of Macrocyclic Disulfide Bridge Peptidomimetics via Bifunctional 5‑Iodo-1,4-Triazoles
Source: J Org Chem. 2026 May 27;91(22):7416–22. doi: 10.1021/acs.joc.6c00195 (PMC13247960; doi:10.1021/acs.joc.6c00195)
Supplement: Supplementary file 1 [file jo6c00195_si_001.pdf]

Supporting Information

On-Resin Synthesis and Diversification of Macrocyclic Disulfide Bridge Peptidomimetics via Bifunctional 5-Iodo-1,4-Triazoles

Michael A. Malone,‡ Oscar A. Shepperson,‡ Jacob E. Simms, Andrew J. Reid and Andrew G. Jamieson*.

[‡] These authors contributed equally.

Corresponding Author

***Andrew G. Jamieson** – *School of Chemistry, Advanced Research Centre, University of Glasgow, 11 Chapel Lane, Glasgow, G11 6EW*; orcid.org/0000-0003-1726-7353; Email: Andrew.jamieson.2@glasgow.ac.uk

Authors

Michael A. Malone – *School of Chemistry, Advanced Research Centre, University of Glasgow, 11 Chapel Lane, Glasgow, G11 6EW*; orcid.org/0009-0007-1476-0659
Oscar A. Shepperson – *School of Chemistry, Advanced Research Centre, University of Glasgow, 11 Chapel Lane, Glasgow, G11 6EW*; orcid.org/0000-0001-7190-8786
Jacob E. Simms – *School of Chemistry, Advanced Research Centre, University of Glasgow, 11 Chapel Lane, Glasgow, G11 6EW*; orcid.org/0009-0000-1812-3576
Andrew J. Reid – *School of Chemistry, Advanced Research Centre, University of Glasgow, 11 Chapel Lane, Glasgow, G11 6EW*; orcid.org/0009-0003-9834-5479

Contents

Supporting Information S1

General Information and Protocols S5

Reagents and Instrumentation S5

General Protocol 1 for the Automated Fmoc-SPPS of C-terminal Amide Peptides S7

General Protocol 2 for the Manual Fmoc-SPPS of C-terminal Acid Peptides S7

General Protocol 3 for On-resin Disubstituted 1,4-triazole Formation by Copper-catalyzed Azide-Alkyne Cycloaddition (CuAAC) S7

General Protocol 4 for Synthesis of On-Resin Trisubstituted 5-Iodo-1,4-Triazole Formation by modified Copper-catalyzed Azide-Alkyne Cycloaddtion (CuAAC) S7

General Protocol 5 for Suzuki Functionalization of On-Resin 5-Iodo-1,4-Triazoles S8

General Protocol 6 for Sonogashira Functionalization of On-Resin 5-Iodo-1,4-Triazoles S8

General Protocol 7 for Resin Cleavage and Global Deprotection S8

Supporting Information Schemes, Tables and Figures S9

Experimental Section S13

Table of Peptides S13

Peptide Synthesis S16

Synthesis of **1e** S16

Synthesis of **1f** S17

Synthesis of **1g** S18

Synthesis of **1h** S19

Synthesis of **1i** S20

Synthesis of **1j** S21

Synthesis of **1k** S22

Synthesis of **2a** S23

Synthesis of **2b** S24

Synthesis of **2c** S25

Synthesis of **3a** S26

Synthesis of **3b** S27

Synthesis of **3c** S28

Synthesis of **3d** S29

Synthesis of **3e** S30

Synthesis of **4a** S31

Synthesis of **4b** S32
Synthesis of **4c** S33
Synthesis of **4d** S34
Synthesis of **4e** S35
Synthesis of **5a** S36
Synthesis of **5b** S37
Synthesis of **5c** S38
Synthesis of **5d** S39
Synthesis of **5e** S40
Characterisation S41
References S54

General Information and Protocols

Reagents and Instrumentation

All reagents were purchased from commercial sources and used without further purification unless otherwise stated. Standard Fmoc-protected amino acids were purchased from CEM Corporation and Pepceuticals, unless specifically stated differently below. Side chain protecting groups of N^{α}-Fmoc amino acids were as follows; Fmoc-Asn(Trt)-OH (Trt = triphenylmethyl), Fmoc-Asp(^{t}Bu)-OH (^{t}Bu = *tert*-butyl), Fmoc-Arg(Pbf)-OH (Pbf = 2,2,4,6,7-pentamethyldihydrobenzofuran-5-sulfonyl), Fmoc-Gln(Trt)-OH, Fmoc-His(Trt)-OH, Fmoc-Lys(Boc)-OH (Boc = *tert*-butyloxycarbonyl), Fmoc-Ser(^{t}Bu)-OH, Fmoc-Thr(^{t}Bu)-OH, Fmoc-Trp(Boc)-OH and Fmoc-Tyr(^{t}Bu)-OH.

N,N-Dimethylformamide (DMF) and diethyl ether (Et_2O) were purchased from Rathburn. Triisopropylsilane (TIPS), *N*-chlorosuccinimide (NCS), di-*tert*-butyl dicarbonate (Boc_2O) and 2-chlorotrityl chloride (2-CTC) resin were purchased from Sigma-Aldrich, Merck. *N,N*-diisopropylethylamine (DIPEA), 1,4-dioxane, 1-[Bis(dimethylamino)methylene]-1*H*-1,2,3-triazolo[4,5-*b*]pyridinium 3-oxide hexafluorophosphate (HATU), palladium-tetrakis(triphenylphosphine) ($Pd(PPh_3)_4$), potassium phosphate trihydrate ($K_3PO_4.3H_2O$), Tris[(1-benzyl-1*H*-1,2,3-triazol-4-yl)methyl]amine (TBTA), *N*-iodosuccinimide (NIS), Copper (I) iodide (CuI), methanol-$_{d4}$ (CD_3OD), 2,2,2-trifluoroethanol (TFE), Fmoc-Aha-OH, Fmoc-Orn(Boc)-OH, Fmoc-Tyr(OEt)-OH, Fmoc-Pra-OH, 4-pentynoic acid (PyA), Fmoc-D-Tyr(^{t}Bu)-OH, caesium carbonate (Cs_2CO_3 [mesh 200]), pyridine, 4-ethynylaniline, 4-ethynylbenzoic acid, 2-methylphenylacetylene, but-3-yn-1-amine, 3-butyn-1-ol, acetic anhydride (Ac_2O), 4-methoxycarbonylphenylboronic acid pinacol ester, and dimethyl sulfoxide (DMSO) were purchased from Fluorochem. Dichloromethane (CH_2Cl_2) was purchased from VWR. Acetonitrile (MeCN), trifluoroacetic acid (TFA), piperidine and formic acid (FA) were purchased from Fisher Scientific. Aminomethyl Rink-Amide resin was purchased from Activotec. 1-Dodecyne was purchased from Thermo Scientific.

Dry solvents were purified using a PureSolv 500 MD solvent purification system.

Analytical reverse-phase high-performance liquid chromatography (RP-HPLC) was performed on a Shimadzu RP-HPLC system with Shimadzu LC-20AT pumps, a Shimadzu SIL20A autosampler and a Shimadzu SPD-20A UV-vis detector, using a Phenomenex Aeris Peptide XB-C18 (100Å, 5 μm, 150 × 4.6 mm). Compounds were eluted with linear gradients at column-dependent flow rates (1 mL/min for the Aeris), where buffer A = 0.1% TFA in H_2O and buffer B = 0.1% TFA in MeCN. Data is reported as column retention time (t_R) in minutes (mins). Crude peptides were purified by preparative RP-HPLC using an Agilent Technologies 1260 Infinity II Preparative LC System (monitoring at 214 nm and 280 nm), with a Phenomenex Gemini column 5 μm C18 column (100 Å, 250 x 21.2 mm). Peptides were eluted on linear gradients (10 mL/min) as determined by analytical RP-HPLC. The solvents employed were buffer; A = H_2O + 0.1% TFA, and buffer; B = MeCN + 0.1% TFA.

Liquid chromatography-mass spectrometry (LCMS) was performed on a Thermo Scientific LCQ Fleet Ion Trap Mass Spectrometer using positive mode electrospray ionization (ESI^+).

Where buffer A = 0.1% TFA in 95% H_2O/5% MeCN and buffer B = 0.1% TFA in 95% MeCN/5% H_2O, a linear gradient of 5% – 95%B over 20 min with a flow rate of 1 mL/min was used with a Phenomenex Aeris Peptide XB-C18 (100 Å, 5 µm, 150 mm x 4.6 mm).

Reactions where heating was required employed a hot plate unless defined otherwise. Where microwave heating was required, it was completed with a CEM Explorer 12 Hybrid Microwave in a sealed reaction vessel with temperature monitoring through a non-contact, infrared sensor.

Nuclear magnetic resonance experiments were performed with a Bruker AVANCE 400 MHz (^1H) spectrometer at 0.5 mM peptide concentration. Chemical shifts are recorded in parts per million (ppm) and were referenced to the residual solvent peak for CD_3OD: δ = 4.78 ppm for ^1H spectra.

Infrared (IR) Spectroscopy was recorded using a Shimadzu FTIR-8400S.

Experiments where conversions are reported were performed as single experiments.

General Protocol 1 for the Automated Fmoc-SPPS of C-terminal Amide Peptides

Peptides were synthesized batchwise as required on an Automated Biotage Syro II microwave synthesizer on a 0.02 mmol scale. The peptides were synthesized using Rink-Amide functionalized Aminomethyl resin (0.62 mmol/g loading, 0.032 g). Peptides were elongated in cycles of amino acid coupling followed by Fmoc removal. Fmoc-protected amino acid (5 equiv., 0.5 M in DMF) coupling was achieved by treatment with Oxyma (5 equiv., 0.5 M in DMF) and DIC (5 equiv., 0.5 M in DMF) at 50 °C for 2 x 60 mins. Fmoc removal was achieved by treatment with 40% piperidine + 5% formic acid in DMF (8 mL, *v/v/v*) at 75 °C for 1 min followed by 75 °C for 5 mins. The resin was washed with DMF following Fmoc removals (4 x 8 mL), and after coupling (2 x 8 mL). Following complete elongation of the peptides, resin aliquots of 0.02 mmol of the respective peptides were combined with one another into homogeneous portions.

General Protocol 2 for the Manual Fmoc-SPPS of C-terminal Acid Peptides

Peptides were synthesized manually on 2-CTC resin (0.25 mmol, 1.087 mmol/g, 0.229 g). Loading of the first amino acid (4 equiv.) was undertaken with DIPEA (2.00 mmol, 258.5 µL, 8 equiv.) in CH_2Cl_2/DMF (95/5, *v/v*) for 2 h at r.t. Following initial loading the resin was capped with a solution of DIPEA and MeOH for 30 mins at r.t. The remaining peptide was elongated with repeating cycles of Fmoc removal followed by amino acid (4 equiv.) coupling employing HATU (361 mg, 0.38 mmol, 3.8 equiv.) and DIPEA (437 uL, 2.5 mmol, 10 equiv.) in DMF for 60 mins at r.t.

General Protocol 3 for On-resin Disubstituted 1,4-triazole Formation by Copper-catalyzed Azide-Alkyne Cycloaddition (CuAAC)

The peptidyl resin (0.05 mmol, 1 equiv.) was swollen in DMF/TFE, 1:1, (*v/v,* 10 mL per 0.1 mmol resin). To this, copper iodide (8.6 mg, 0.045 mmol, 0.9 equiv.), DIPEA (43.7 µL, 0.25 mmol, 5 equiv.), 2,6-lutidine (28.9 µL, 0.25 mmol, 5 equiv.), NaAsc (14.9 mg, 0.075 mmol, 1.5 equiv.) and TBTA (31.8 mg, 0.06 mmol, 1.2 equiv.) were added and the reaction heated (10 – 120 mins, 55 °C, µW). Upon reaction completion, the resin was washed with DMF (3 x 5 mL), CH_2Cl_2 (3 x 5 mL), and dried under vacuum.[1]

General Protocol 4 for Synthesis of On-Resin Trisubstituted 5-Iodo-1,4-Triazole Formation by modified Copper-catalyzed Azide-Alkyne Cycloaddtion (CuAAC)

The peptidyl resin (0.05 mmol, equiv.) was swollen in DMF (10 mL final volume per 0.1 mmol resin) with DIPEA (17.4 uL, 0.10 mmol, 0.1 equiv.) for 5 min prior to the addition of any further reagents. CuI (11.4 mg, 0.06 mmol, 1.2 equiv.) in DMF and NBS (9.8 mg, 0.055 mmol, 1.1 equiv.) in DMF were solubilized separately in equal volumes of DMF and combined immediately prior to addition to the peptidyl resin. The reaction was mixed 12 h at r.t. Upon reaction completion and following Fmoc removal (20% piperidine + 5% formic acid in DMF, 2 x 5 mL), the peptidyl resin was washed with DMF (3 x 5 mL), 20% piperidine + 5% formic acid in DMF (2 x 5 mL, *v/v/v*), DMF (3 x 5 mL), CH_2Cl_2 (3 x 5 mL), and dried under vacuum.[1]

General Protocol 5 for Suzuki Functionalization of On-Resin 5-Iodo-1,4-Triazoles

The peptidyl resin was swollen in 1,4-dioxane (5 mL per 0.1 mmol resin) for 5 minutes prior to the addition of any further reagents. Following resin swelling, $K_3PO_4.3H_2O$ (66.6 mg, 0.25 mmol, 5 equiv.) in H_2O (5% *v/v* of total final volume) and 4-methoxycarbonylphenylboronic acid pinacol ester (131.1 mg, 0.5 mmol, 10 equiv.) in 1,4-dioxane (2 mL) were added to the solution. The resin containing solution was degassed with argon (10 mins) prior to reaction initiation by addition of the respective $Pd(PPh_3)_4$ (5.8 mg, 0.005 mmol, 10 mol%) at 60 °C for 1 – 18 hours. Upon reaction completion, the peptidyl resin was washed with DMF (3 x 5 mL), 20% piperidine + 5% formic acid in DMF (2 x 5 mL, *v/v/v*), DMF (3 x 5 mL), CH_2Cl_2 (3 x 5 mL), and dried under vacuum.[1]

General Protocol 6 for Sonogashira Functionalization of On-Resin 5-Iodo-1,4-Triazoles

The resin-bound peptide was swollen in DMF (5 mL per 0.1 mmol resin) for 5 minutes prior to the addition of any further reagents. Following resin swelling, DIPEA (43.5 µL, 0.25 mmol, 5 equiv.), CuI (1.9 mg, 0.1 mmol, 0.2 equiv.) in H_2O (10% *v/v* of total final volume) and phenylacetylene (51 mg, 0.5 mmol, 10 equiv.) were added to the solution. The resin containing solution was degassed with argon (10 mins) prior to reaction initiation by addition of the respective $Pd(PPh_3)_4$ (5.8 mg, 0.005 mmol, 10 mol%) at 70 °C for 4 hours. Upon reaction completion, the peptidyl resin was washed with DMF (3 x 5 mL), 20% piperidine + 5% formic acid in DMF (2 x 5 mL, *v/v/v*), DMF (3 x 5 mL), CH_2Cl_2 (3 x 5 mL), and dried under vacuum.

General Protocol 7 for Resin Cleavage and Global Deprotection

The resin-bound peptide was treated with a cleavage cocktail of TFA/H_2O/TIPS (95/2.5/2.5, *v/v/v*, 10 mL per 0.1 mmol resin) and agitated (1 – 2 h, r.t.). The cleavage solution was separated from the resin and its volume reduced under a flow of N_2. Ice cold Et_2O (15 mL per 0.1 mmol resin) was used to precipitate the peptide. The precipitate was isolated by centrifugation, washed once more with ice cold Et_2O, dissolved in MeCN/MQ H_2O (2:8, *v/v*) and analyzed by RP-HPLC and LCMS. Following determination of the desired peptidyl products, the solutions were frozen in $N_{2(l)}$ and lyophilized.

Supporting Information Schemes, Tables and Figures

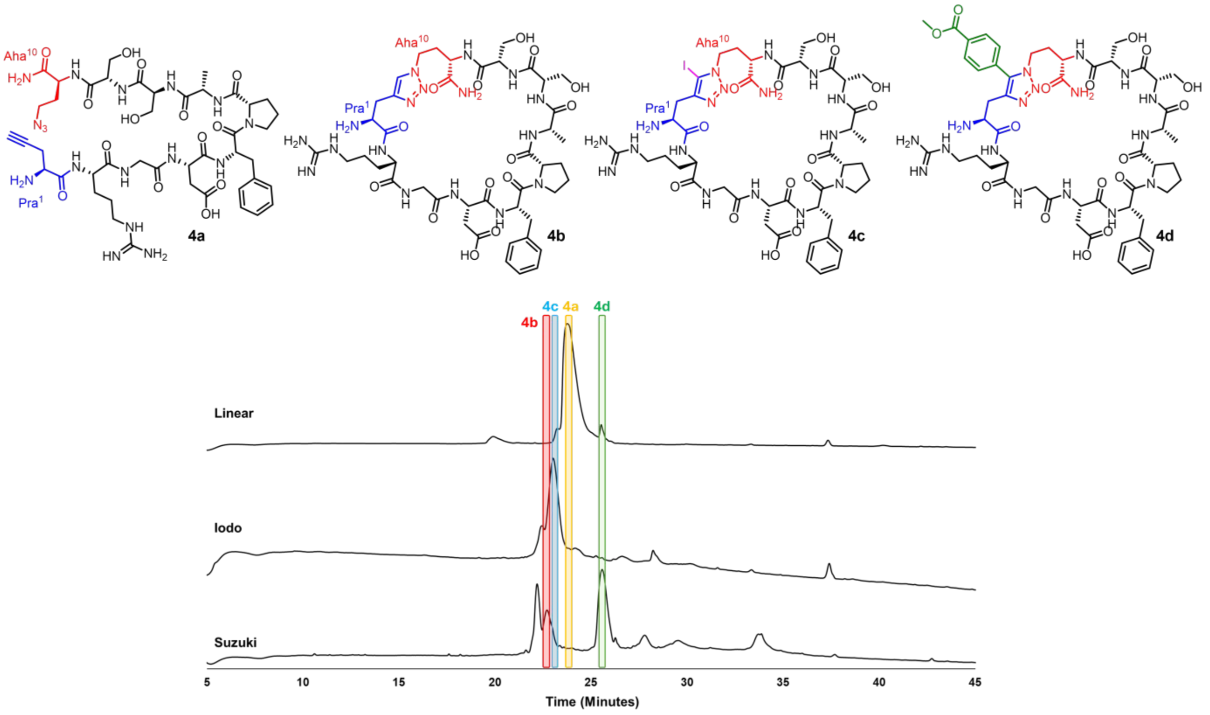

Supporting Information Figure S1. Analytical RP-HPLC chromatogram (214 nm) for reaction monitoring for synthesis of peptide **4d**. Reaction overview comparing a) linear **4a**; b) iodo **4c**; c) Suzuki product **4d**, of peptide 4 respectively. **RP-HPLC:** linear gradient = 0% – 50%B over 50 min, flow rate = 1.0 mL/min, l = 214 nm; t_R = 23.8 min (minor = **4a, yellow**), 22.4 min (minor = **4b, red**), 23.1 min (minor = **4c, blue**) and 25.6 min (major = **4d, green**).

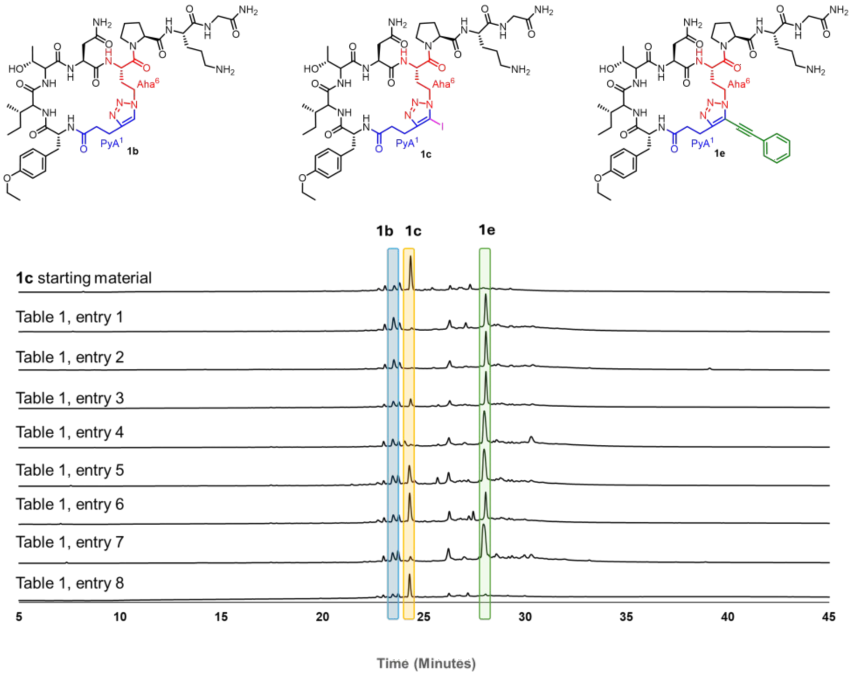

Supporting Information Figure S2. Analytical RP-HPLC chromatogram (214 nm) for entries 1 – 8 of Sonogashira optimization table, with the formation of peptide **1e**. **RP-HPLC:** linear gradient = 5% – 95%B over 60 min, flow rate = 1.0 mL/min, l = 214 nm; t_R = 24.3 min (minor = **1b**), 24.6 min (minor = **1c**) and 28.3 min (major = **1e**).

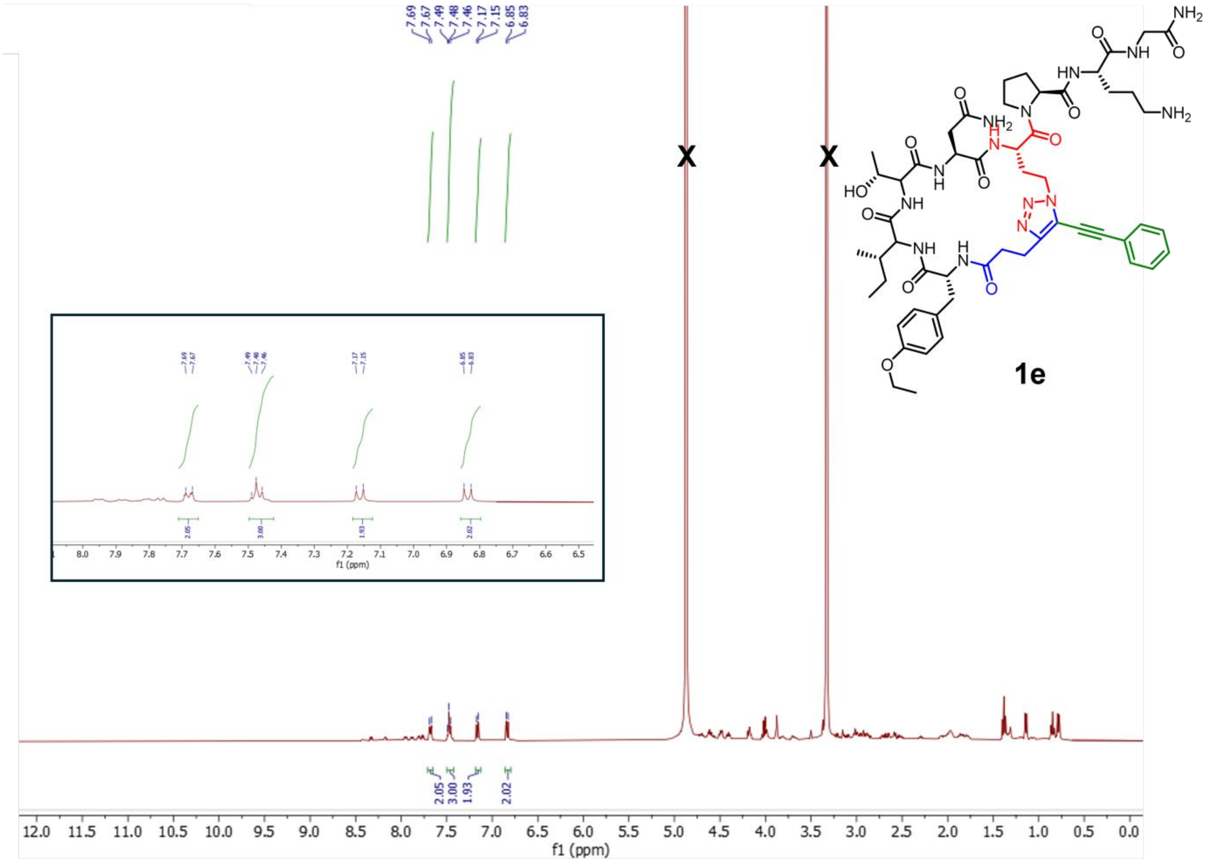

Supporting Information Figure S3. ^{1}H NMR (400 MHz, 298 K, CD_3OD) spectra of the peptide **1e** with amide region of interest inset. Spectrum referenced to CD_3OD 4.78 ppm. X's denote solvent peaks for CD_3OD and H_2O.

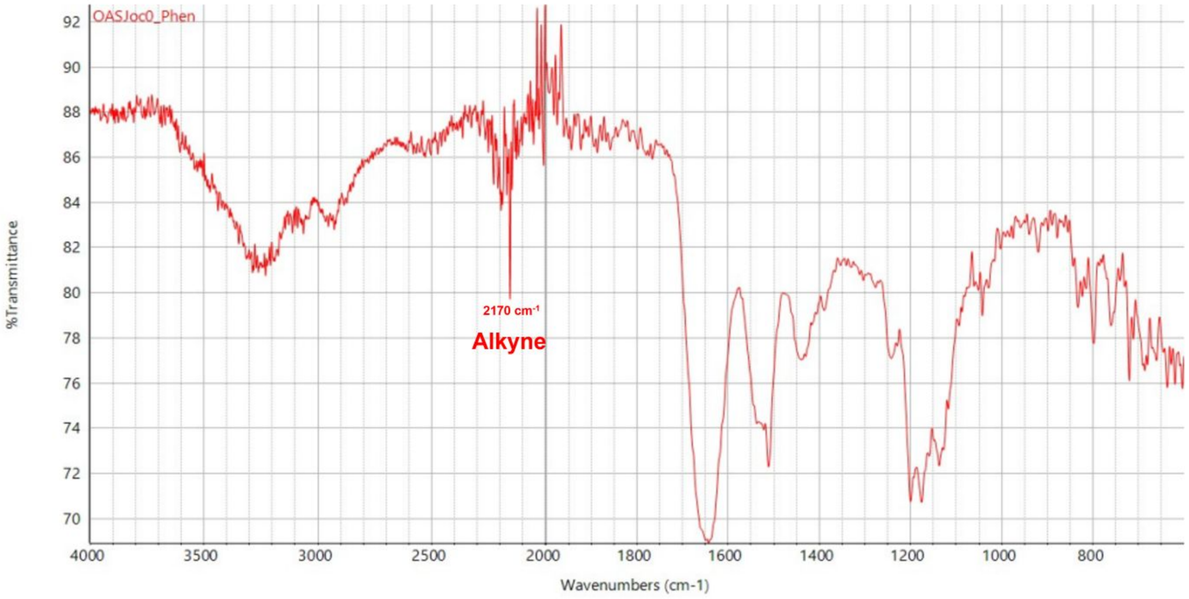

Supporting Information Figure S4. Raman IR spectra of the peptide **1e**.

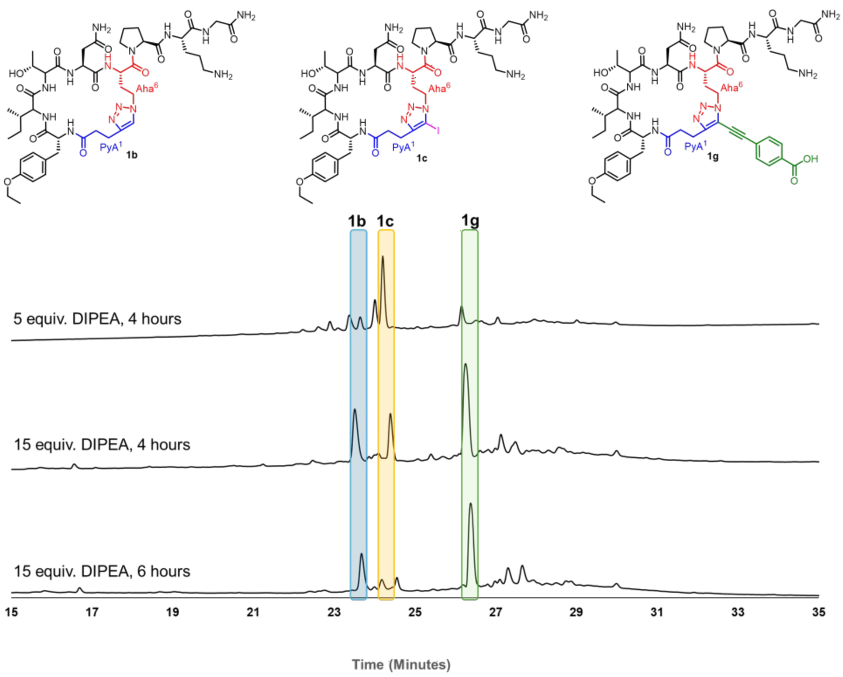

Supporting Information Figure S5. Analytical RP-HPLC chromatogram (214 nm) of peptide **1g**. Reaction overview comparing a) 5 equiv. of DIPEA; b) 15 equiv. of DIPEA (4 h), c) 15 equiv. of DIPEA (6 h). **RP-HPLC:** linear gradient = 5% – 95%B over 60 min, flow rate = 1.0 mL/min, l = 214 nm; t_R = 24.3 min (minor = **1b**), 24.6 min (minor = **1c**) and 26.3 min (major = **1g**).

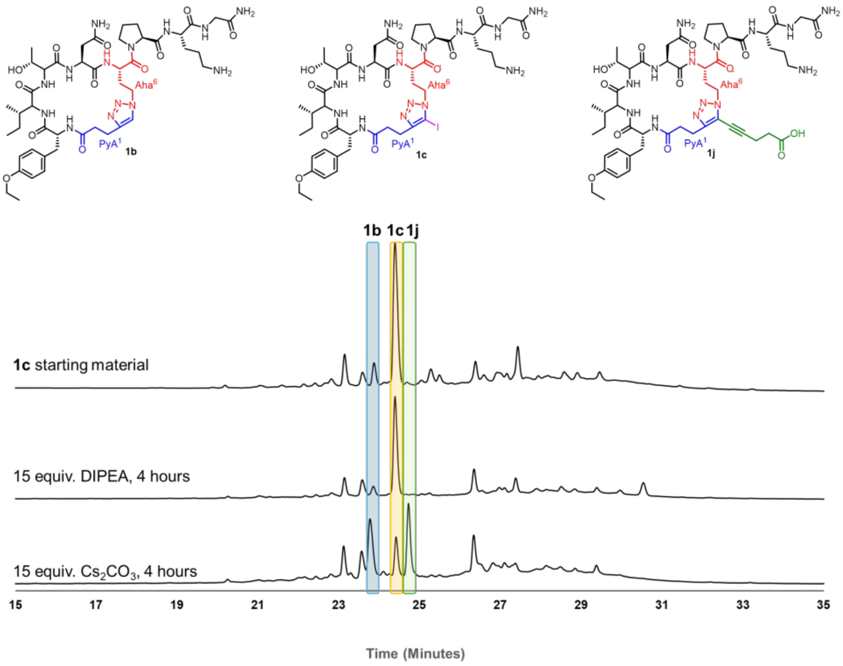

Supporting Information Figure S6. Analytical RP-HPLC chromatogram (214 nm) of peptide **1j**. Reaction overview comparing a) 5 equiv. of DIPEA; b) 15 equiv. of DIPEA; c) 15 equiv. of Cs_2CO_3. **RP-HPLC:** linear gradient = 5% – 95%B over 60 min, flow rate = 1.0 mL/min, l = 214 nm; t_R = 24.3 min (minor = **1b**), 24.6 min (minor = **1c**) and 24.7 min (major = **1j**).

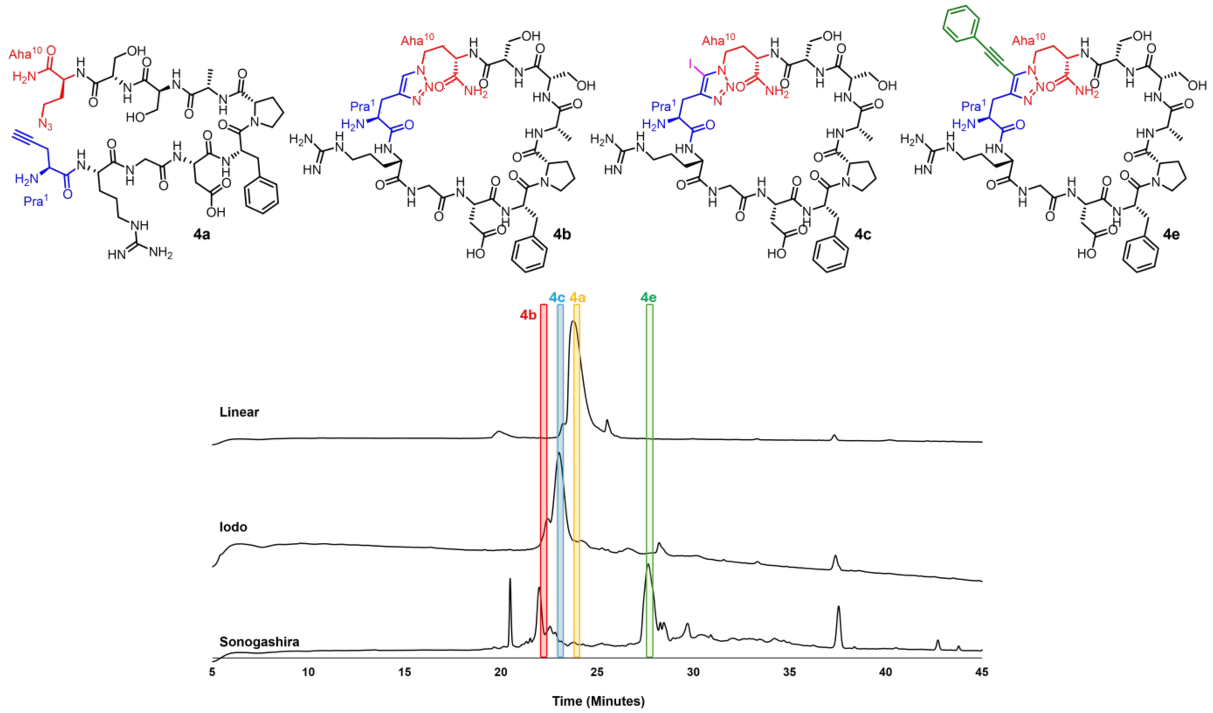

Supporting Information Figure S7. Analytical RP-HPLC chromatogram (214 nm) for reaction monitoring for synthesis of peptide **4e**. Reaction overview comparing a) linear **4a**; b) iodo **4c**; c) Sonogashira product **4e**, of peptide 4 respectively. **RP-HPLC:** linear gradient = 0% – 50%B over 50 min, flow rate = 1.0 mL/min, l = 214 nm; t_R = 23.8 min (minor = **4a, yellow**), 22.4 min (minor = **4b, red**), 23.1 min (minor = **4c, blue**) and 27.7 min (major = **4e, green**).

Experimental Section

Table of Peptides

Supporting Information Table S1. Name, sequence, % yield, % purity, *m/z* and retention time of peptides. Abbreviations: Aha =Azidohomoalanine, OH = C-terminal acid, NH_2 = C-terminal amide, Orn = Ornithine, Pra = Propargylglycine, PyA = 4-Pentynoic Acid and y-(OEt) = D-Tyrosine(OEt). Detailed characterization data for peptides can be found in **Supporting Information Figures S6 – S30**.

Peptide	Linear Sequence	Bridge – 5'-Functionality	Yield (%)	% Purity	Calculated *m/z*	Observed *m/z*	t_R (mins)
**1e**	*(PyA)(y-OEt)ITN(Aha)P(Orn)G-NH$_2$*	Triazole – Phenylacetylene	2	96	1111.27	1111.43 ± 0.45	28.3
**1f**	*(PyA)(y-OEt)ITN(Aha)P(Orn)G-NH$_2$*	Triazole – Aniline Alkyne	4	99	1126.29	1126.18 ± 0.25	24.4
**1g**	*(PyA)(y-OEt)ITN(Aha)P(Orn)G-NH$_2$*	Triazole – Benzoic Alkyne	5	99	1155.28	1155.28 ± 0.25	26.3
**1h**	*(PyA)(y-OEt)ITN(Aha)P(Orn)G-NH$_2$*	Triazole – Amino Alkyne	4	95	1078.24	1077.94 ± 0.43	22.9
**1i**	*(PyA)(y-OEt)ITN(Aha)P(Orn)G-NH$_2$*	Triazole – Alcohol Alkyne	5	95	1079.23	1079.14 ± 0.31	24.3
**1j**	*(PyA)(y-OEt)ITN(Aha)P(Orn)G-NH$_2$*	Triazole – Acid Alkyne	2	94	1107.24	1106.68 ± 0.21	24.7
**1k**	*(PyA)(y-OEt)ITN(Aha)P(Orn)G-NH$_2$*	Triazole – Lipid Alkyne	3	98	1175.44	1175.16 ± 0.42	36.4
**2a**	*H-(Pra)HIQN(Aha)PLG-NH$_2$*	N/A (linear)	24	97	998.12	998.10 ± 0.21	19.0
**2b**	*H-(Pra)HIQN(Aha)PLG-NH$_2$*	Triazole – H	1	98	998.12	998.24 ± 0.35	17.2

**2c**	*H-(Pra)HIQN(Aha)PLG-NH$_2$*	Triazole – I	1	92	1124.01	1123.60 ± 0.34	19.1
**3a**	*[(Pra)IEGSPV(Aha)FPDG]*	N/A (linear)	1	95	1220.31	1220.09 ± 0.11	25.3
**3b**	*[(Pra)IEGSPV(Aha)FPDG]*	Triazole – H	1	98	1220.31	1219.97 ± 0.26	23.7
**3c**	*[(Pra)IEGSPV(Aha)FPDG]*	Triazole – I	1	99	1346.21	1345.55 ± 0.06	25.1
**3d**	*Fmoc-(Pra)IEGSPV(Aha)FPDG-OH*	Triazole – Benzylmethylester	1	96	1594.70	1594.47 ± 0.10	33.4
**3e**	*Fmoc-(Pra)IEGSPV(Aha)FPDG-OH*	Triazole – Phenylacetylene	1	95	1560.69	1560.55 ± 0.02	34.5
**4a**	*H-(Pra)RGDFPASS(Aha)-NH$_2$*	N/A (linear)	12	99	1056.11	1056.11 ± 0.18	17.7
**4b**	*H-(Pra)RGDFPASS(Aha)-NH$_2$*	Triazole – H	8	99	1056.11	1056.07 ± 0.22	16.7
**4c**	*H-(Pra)RGDFPASS(Aha)-NH$_2$*	Triazole – I	1	98	1182.01	1181.85 ± 0.36	17.0
**4d**	*H-(Pra)RGDFPASS(Aha)-NH$_2$*	Triazole – Benzylmethylester	1	99	1190.24	1189.91 ± 0.64	18.6
**4e**	*H-(Pra)RGDFPASS(Aha)-NH$_2$*	Triazole – Phenylacetylene	1	98	1156.23	1155.95 ± 0.47	20.6
**5a**	*H-AG(Pra)KNFFWKTFTS(Aha)-NH$_2$*	N/A (linear)	8	98	1695.91	1695.32 ± 0.80	27.4
**5b**	*H-AG(Pra)KNFFWKTFTS(Aha)-NH$_2$*	Triazole – H	1	98	1695.91	1695.71 ± 0.21	25.5
**5c**	*H-AG(Pra)KNFFWKTFTS(Aha)-NH$_2$*	Triazole – I	1	95	1821.80	1821.69 ± 0.22	26.0
**5d**	*H-AG(Pra)KNFFWKTFTS(Aha)-NH$_2$*	Triazole – Benzylmethylester	1	96	1830.04	1830.04	26.4

**5e**	*H-AG(Pra)KNFFWKTFTS(Aha)-*NH_2	Triazole – Phenylacetylene	1	95	1796.03	1796.03	27.2

Peptide Synthesis

Synthesis of **1e**

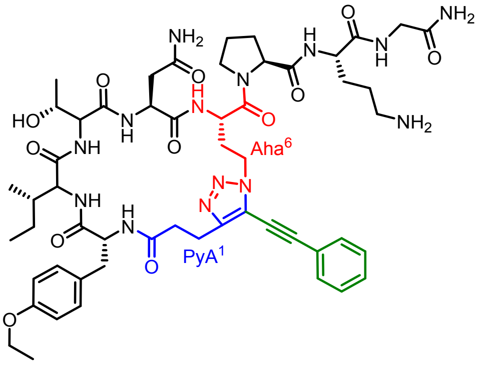

The linear precursor for peptide **1e** was synthesized by automated Fmoc-SPPS as described in **General Protocol 1** with PyA and Aha incorporated at positions 1 and 6 respectively. Following synthesis of the linear peptide, the peptidyl resin (0.05 mmol) was subjected to trisubstituted copper-catalyzed azide-alkyne cycloaddition (CuAAC) conditions, as described in **General Protocol 4**. The cyclized peptide underwent an on-resin Sonogashira coupling with phenylacetylene as described by **General Method 6**. The final cyclized peptide was liberated from the resin and its protecting groups simultaneously removed under the conditions described in **General Protocol 7** to afford the crude cyclized peptide, **1e** (25.2 mg, 45% yield [based on initial resin loading]) at approximately 26% purity.

Crude peptide **1e** (25.2 mg), was solubilized in a solution of 0.1% TFA in MeCN:MQ H_2O (2:8; *v/v*) at a concentration of ~25 mg/mL and purified by preparative RP-HPLC (1 x 1000 µL injection) employing a gradient of 15% – 60%B over 45 min (*ca.* 1%B/min) at a flow rate of 10 mL/min. Fractions were analyzed by RP-HPLC and LCMS for compound identification and lyophilized to afford the compound, **1e**, as a white amorphous powder (0.9 mg, 8% recovery [based on crude yield], 96% purity, 2% overall yield).

LCMS (ESI+): Mass calculated for $[C_{54}H_{74}N_{14}O_{12} + H]$ 1111.27; deconvoluted mass observed 1111.43 ± 0.45. Charge states; 556.88 $[M+2H]^{2+}$, 1112.10 $[M+H]^{+}$.

RP-HPLC: linear gradient = 5% – 95%B over 60 min, flow rate = 1.0 mL/min, l = 214 nm; t_R = 28.3 min.

Synthesis of **1f**

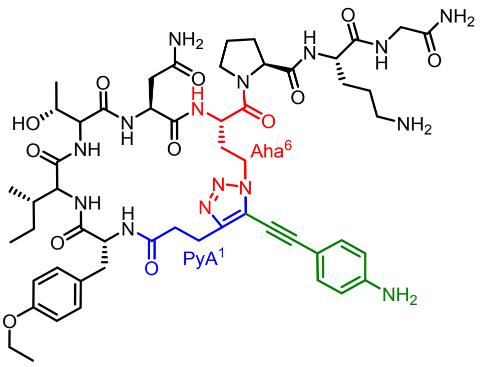

The linear precursor for peptide **1f** was synthesized by automated Fmoc-SPPS as described in **General Protocol 1** with PyA and Aha incorporated at positions 1 and 6 respectively. Following synthesis of the linear peptide, the peptidyl resin (0.05 mmol) was subjected to trisubstituted copper-catalyzed azide-alkyne cycloaddition (CuAAC) conditions, as described in **General Protocol 4**. The cyclized peptide underwent an on-resin Sonogashira coupling with 4-ethynylaniline as described by **General Method 6**. The final cyclized peptide was liberated from the resin and its protecting groups simultaneously removed under the conditions described in **General Protocol 7** to afford the crude cyclized peptide, **1f** (26.8 mg, 48% yield [based on initial resin loading]) at approximately 47% purity.

Crude peptide **1f** (47 mg), was solubilized in a solution of 0.1% TFA in MeCN:MQ H_2O (2:8; *v/v*) at a concentration of ~25 mg/mL and purified by preparative RP-HPLC (1 x 1900 μL injection) employing a gradient of 15% – 60%B over 45 min (*ca.* 1%B/min) at a flow rate of 10 mL/min. Fractions were analyzed by RP-HPLC and LCMS for compound identification and lyophilized to afford the compound, **1f**, as a white amorphous powder (2.3 mg, 4% recovery [based on crude yield], 99% purity, 9% overall yield).

LCMS (ESI+): Mass calculated for [$C_{54}H_{75}N_{15}O_{12}$ + H] 1126.29; deconvoluted mass observed 1126.18 ± 0.25. Charge states; 564.18 $[M+2H]^{2+}$, 1127.00 $[M+H]^{+}$.

RP-HPLC: linear gradient = 5% – 95%B over 60 min, flow rate = 1.0 mL/min, l = 214 nm; t_R = 24.4 min.

Synthesis of **1g**

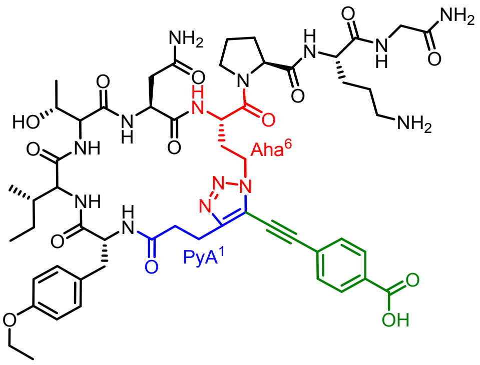

The linear precursor for peptide **1g** was synthesized by automated Fmoc-SPPS as described in **General Protocol 1** with PyA and Aha incorporated at positions 1 and 6 respectively. Following synthesis of the linear peptide, the peptidyl resin (0.05 mmol) was subjected to trisubstituted copper-catalyzed azide-alkyne cycloaddition (CuAAC) conditions, as described in **General Protocol 4**. The cyclized peptide underwent an on-resin Sonogashira coupling with 4-ethynylbenzoic acid as described by **General Method 6**. The final cyclized peptide was liberated from the resin and its protecting groups simultaneously removed under the conditions described in **General Protocol 7** to afford the crude cyclized peptide, **1g** (52.1 mg, 90% yield [based on initial resin loading]) at approximately 30% purity.

Crude peptide **1g** (52.1 mg), was solubilized in a solution of 0.1% TFA in MeCN:MQ H_2O (2:8; *v/v*) at a concentration of ~25 mg/mL and purified by preparative RP-HPLC (1 x 2000 µL injection) employing a gradient of 15% – 60%B over 45 min (*ca.* 1%B/min) at a flow rate of 10 mL/min. Fractions were analyzed by RP-HPLC and LCMS for compound identification and lyophilized to afford the compound, **1g**, as a white amorphous powder (2.6 mg, 5% recovery [based on crude yield], 99% purity, 5% overall yield).

LCMS (ESI+): Mass calculated for [$C_{55}H_{74}N_{14}O_{14}$ + H] 1155.28; deconvoluted mass observed 1155.28 ± 0.25. Charge states; 578.73 $[M+2H]^{2+}$, 1156.10 $[M+H]^{+}$.

RP-HPLC: linear gradient = 5% – 95%B over 60 min, flow rate = 1.0 mL/min, l = 214 nm; t_R = 26.3 min.

Synthesis of **1h**

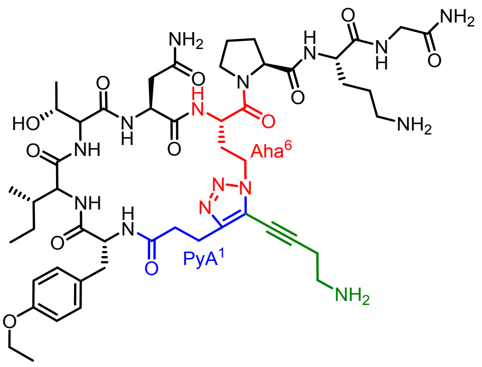

The linear precursor for peptide **1h** was synthesized by automated Fmoc-SPPS as described in **General Protocol 1** with PyA and Aha incorporated at positions 1 and 6 respectively. Following synthesis of the linear peptide, the peptidyl resin (0.025 mmol) was subjected to trisubstituted copper-catalyzed azide-alkyne cycloaddition (CuAAC) conditions, as described in **General Protocol 4**. The cyclized peptide underwent an on-resin Sonogashira coupling with but-3-yn-1-amine as described by **General Method 6**. The final cyclized peptide was liberated from the resin and its protecting groups simultaneously removed under the conditions described in **General Protocol 7** to afford the crude cyclized peptide, **1h** (19 mg, 73% yield [based on initial resin loading]) at approximately 40% purity.

Crude peptide **1h** (19 mg), was solubilized in a solution of 0.1% TFA in MeCN:MQ H_2O (2:8; *v/v*) at a concentration of ~10 mg/mL and purified by preparative RP-HPLC (2 x 1000 µL injection) employing a gradient of 15% – 60%B over 45 min (*ca.* 1%B/min) at a flow rate of 10 mL/min. Fractions were analyzed by RP-HPLC and LCMS for compound identification and lyophilized to afford the compound, **1h**, as a white amorphous powder (1.0 mg, 5% recovery [based on crude yield], 95% purity, 4% overall yield).

LCMS (ESI+): Mass calculated for [$C_{50}H_{75}N_{15}O_{12}$ + H] 1078.24; deconvoluted mass observed 1077.94 ± 0.43. Charge states; 540.12 $[M+2H]^{2+}$, 1078.63 $[M+H]^{+}$.

RP-HPLC: linear gradient = 5% – 95%B over 60 min, flow rate = 1.0 mL/min, l = 214 nm; t_R = 22.9 min.

Synthesis of **1i**

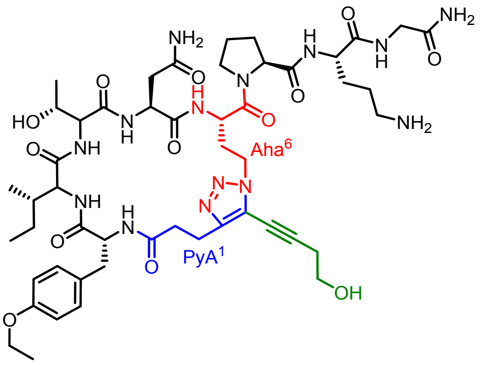

The linear precursor for peptide **1i** was synthesized by automated Fmoc-SPPS as described in **General Protocol 1** with PyA and Aha incorporated at positions 1 and 6 respectively. Following synthesis of the linear peptide, the peptidyl resin (0.05 mmol) was subjected to trisubstituted copper-catalyzed azide-alkyne cycloaddition (CuAAC) conditions, as described in **General Protocol 4**. The cyclized peptide underwent an on-resin Sonogashira coupling with 3-butyn-1-ol as described by **General Method 6**. The final cyclized peptide was liberated from the resin and its protecting groups simultaneously removed under the conditions described in **General Protocol 7** to afford the crude cyclized peptide, **1i** (32.2 mg, 60% yield [based on initial resin loading]) at approximately 62% purity.

Crude peptide **1i** (32.2 mg), was solubilized in a solution of 0.1% TFA in MeCN:MQ H_2O (2:8; *v/v*) at a concentration of ~20 mg/mL and purified by preparative RP-HPLC (1 x 1500 μL injection) employing a gradient of 15% – 60%B over 45 min (*ca.* 1%B/min) at a flow rate of 10 mL/min. Fractions were analyzed by RP-HPLC and LCMS for compound identification and lyophilized to afford the compound, **1i**, as a white amorphous powder (2.7 mg, 8% recovery [based on crude yield], 95% purity, 5% overall yield).

LCMS (ESI+): Mass calculated for [$C_{54}H_{74}N_{14}O_{13}$ + H] 1079.24; deconvoluted mass observed 1079.14 ± 0.31. Charge states; 540.68 $[M+2H]^{2+}$, 1079.91 $[M+H]^{+}$.

RP-HPLC: linear gradient = 5% – 95%B over 60 min, flow rate = 1.0 mL/min, l = 214 nm; t_R = 24.3 min.

Synthesis of **1j**

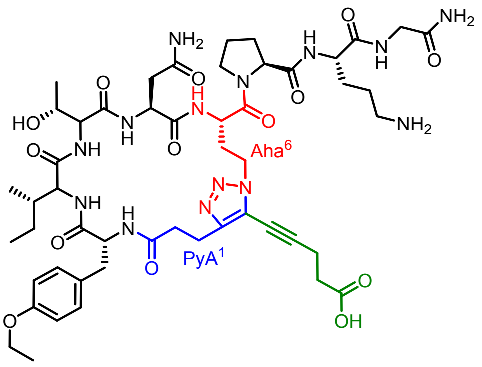

The linear precursor for peptide **1j** was synthesized by automated Fmoc-SPPS as described in **General Protocol 1** with PyA and Aha incorporated at positions 1 and 6 respectively. Following synthesis of the linear peptide, the peptidyl resin (0.075 mmol) was subjected to trisubstituted copper-catalyzed azide-alkyne cycloaddition (CuAAC) conditions, as described in **General Protocol 4**. The cyclized peptide underwent an on-resin Sonogashira coupling with 5-pentynoic acid as described by **General Method 6**. The final cyclized peptide was liberated from the resin and its protecting groups simultaneously removed under the conditions described in **General Protocol 7** to afford the crude cyclized peptide, **1j** (35 mg, 42% yield [based on initial resin loading]) at approximately 35% purity.

Crude peptide **1j** (35 mg), was solubilized in a solution of 0.1% TFA in MeCN:MQ H_2O (2:8; *v/v*) at a concentration of ~17 mg/mL and purified by preparative RP-HPLC (2 x 1000 µL injection) employing a gradient of 15% – 60%B over 45 min (*ca.* 1%B/min) at a flow rate of 10 mL/min. Fractions were analyzed by RP-HPLC and LCMS for compound identification and lyophilized to afford the compound, **1j**, as a white amorphous powder (2.0 mg, 6% recovery [based on crude yield], 94% purity, 2% overall yield).

LCMS (ESI+): Mass calculated for $[C_{51}H_{74}N_{14}O_{14} + H]$ 1107.24; deconvoluted mass observed 1106.68 ± 0.21. Charge states; 554.41 $[M+2H]^{2+}$, 1107.53 $[M+H]^{+}$.

RP-HPLC: linear gradient = 5% – 95%B over 60 min, flow rate = 1.0 mL/min, l = 214 nm; t_R = 24.7 min.

Synthesis of **1k**

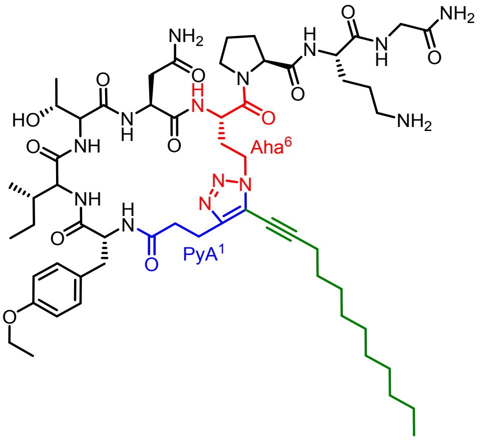

The linear precursor for peptide **1k** was synthesized by automated Fmoc-SPPS as described in **General Protocol 1** with PyA and Aha incorporated at positions 1 and 6 respectively. Following synthesis of the linear peptide, the peptidyl resin (0.05 mmol) was subjected to trisubstituted copper-catalyzed azide-alkyne cycloaddition (CuAAC) conditions, as described in **General Protocol 4**. The cyclized peptide underwent an on-resin Sonogashira coupling with 1-dodecyne as described by **General Method 6**. The final cyclized peptide was liberated from the resin and its protecting groups simultaneously removed under the conditions described in **General Protocol 7** to afford the crude cyclized peptide, **1k** (27 mg, 46% yield [based on initial resin loading]) at approximately 30% purity.

Crude peptide **1k** (57 mg), was solubilized in a solution of 0.1% TFA in MeCN:MQ H_2O (2:8; *v/v*) at a concentration of ~20 mg/mL and purified by preparative RP-HPLC (2 x 1500 µL injection) employing a gradient of 20% – 80%B over 60 min (*ca.* 1%B/min) at a flow rate of 10 mL/min. Fractions were analyzed by RP-HPLC and LCMS for compound identification and lyophilized to afford the compound, **1k**, as a white amorphous powder (1.6 mg, 6% recovery [based on crude yield], 98% purity, 3% overall yield).

LCMS (ESI+): Mass calculated for [$C_{58}H_{90}N_{14}O_{12}$ + H] 1175.44; deconvoluted mass observed 1175.16 ± 0.42. Charge states; 588.73 $[M+2H]^{2+}$, 1175.86 $[M+H]^{+}$.

RP-HPLC: linear gradient = 5% – 95%B over 60 min, flow rate = 1.0 mL/min, l = 214 nm; t_R = 36.4 min.

Synthesis of **2a**

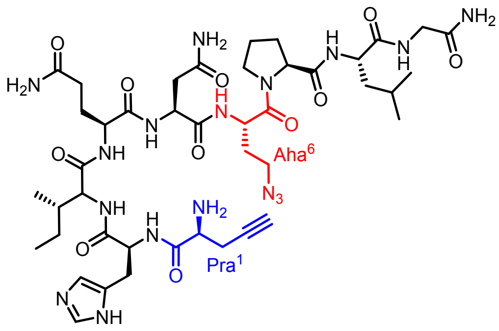

The linear peptide **2a** was synthesized by automated Fmoc-SPPS as described in **General Protocol 1**. The final peptide (0.05 mmol) with Pra and Aha incorporated at positions 1 and 6 respectively was liberated from the resin and its protecting groups simultaneously removed under the conditions described in **General Protocol 7** to afford the crude peptide, **2a** (17.6 mg, 35% yield [based on initial resin loading]) at approximately 85% purity.

Crude peptide **2a** (17 mg), was solubilized in a solution of 0.1% TFA in MeCN:MQ H_2O (2:8; *v/v*) at a concentration of ~17 mg/mL and purified by preparative RP-HPLC (1 x 1000 μL injection) employing a gradient of 15% – 60%B over 45 min (*ca.* 1%B/min) at a flow rate of 10 mL/min. Fractions were analyzed by RP-HPLC and LCMS for compound identification and lyophilized to afford the compound, **2a**, as a white amorphous powder (11.8 mg, 67% recovery [based on crude yield], 97% purity, 24% overall yield).

LCMS (ESI+): Mass calculated for [$C_{43}H_{67}N_{17}O_{11}$ + H] 998.12; deconvoluted mass observed 998.10 ± 0.21. Charge states; 500.12 $[M+2H]^{2+}$, 998.95 $[M+H]^{+}$.

RP-HPLC: linear gradient = 5% – 95%B over 60 min, flow rate = 1.0 mL/min, l = 214 nm; t_R = 19.0 min.

Synthesis of **2b**

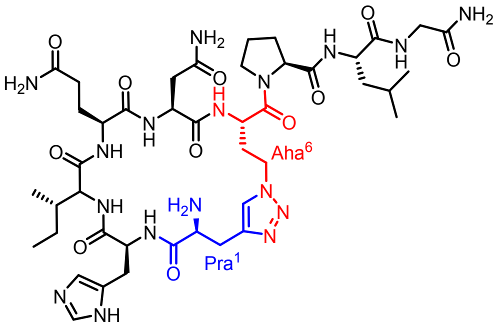

The linear precursor for peptide **2b** was synthesized by automated Fmoc-SPPS as described in **General Protocol 1** with Pra and Aha incorporated at positions 1 and 6 respectively. Following synthesis of the linear peptide, the peptidyl resin (0.05 mmol) was subjected to disubstituted copper-catalyzed azide-alkyne cycloaddition (CuAAC) conditions, as described in **General Protocol 3**. The final cyclized peptide was liberated from the resin and its protecting groups simultaneously removed under the conditions described in **General Protocol 7** to afford the crude cyclized peptide, **2b** (21.5 mg, 43% yield [based on initial resin loading]) at approximately 35% purity.

Crude peptide **2b** (21.5 mg), was solubilized in a solution of 0.1% TFA in MeCN:MQ H_2O (2:8; *v/v*) at a concentration of ~20 mg/mL and purified by preparative RP-HPLC (1 x 1200 µL injection) employing a gradient of 15% – 60%B over 45 min (*ca.* 1%B/min) at a flow rate of 10 mL/min. Fractions were analyzed by RP-HPLC and LCMS for compound identification and lyophilized to afford the compound, **2b**, as a white amorphous powder (1.6 mg, 3% recovery [based on crude yield], 98% purity, 1% overall yield).

LCMS (ESI+): Mass calculated for [$C_{43}H_{67}N_{17}O_{11}$ + H] 998.12; deconvoluted mass observed 998.24 ± 0.35. Charge states; 500.24 $[M+2H]^{2+}$, 998.99 $[M+H]^{+}$.

RP-HPLC: linear gradient = 5% – 95%B over 60 min, flow rate = 1.0 mL/min, l = 214 nm; t_R = 17.2 min.

Synthesis of **2c**

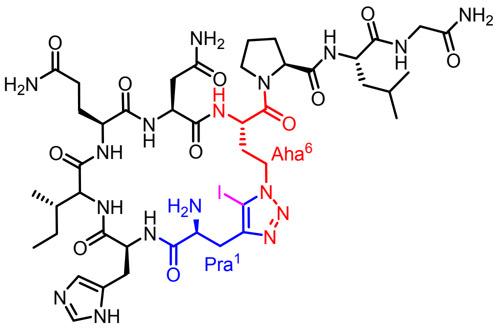

The linear precursor for peptide **2c** was synthesized by automated Fmoc-SPPS as described in **General Protocol 1** with Pra and Aha incorporated at positions 1 and 6 respectively. Following synthesis of the linear peptide, the peptidyl resin (0.05 mmol) was subjected to trisubstituted copper-catalyzed azide-alkyne cycloaddition (CuAAC) conditions, as described in **General Protocol 4**. The final cyclized peptide was liberated from the resin and its protecting groups simultaneously removed under the conditions described in **General Protocol 7** to afford the crude cyclized peptide, **2c** (29.2 mg, 52% yield [based on initial resin loading]) at approximately 55% purity.

Crude peptide **2c** (29.2 mg), was solubilized in a solution of 0.1% TFA in MeCN:MQ H_2O (2:8; *v/v*) at a concentration of ~8 mg/mL and purified by preparative RP-HPLC (1 x 2000 µL injection) employing a gradient of 15% – 60%B over 45 min (*ca.* 1%B/min) at a flow rate of 10 mL/min. Fractions were analyzed by RP-HPLC and LCMS for compound identification and lyophilized to afford the compound, **2c**, as a white amorphous powder (0.5 mg, 2% recovery [based on crude yield], 92% purity, 1% overall yield).

LCMS (ESI+): Mass calculated for [$C_{43}H_{66}IN_{17}O_{11}$ + H] 1124.01; deconvoluted mass observed 1123.60 ± 0.34. Charge states; 562.92 $[M+2H]^{2+}$, 1124.36 $[M+H]^{+}$.

RP-HPLC: linear gradient = 5% – 95%B over 60 min, flow rate = 1.0 mL/min, l = 214 nm; t_R = 19.1 min.

Synthesis of **3a**

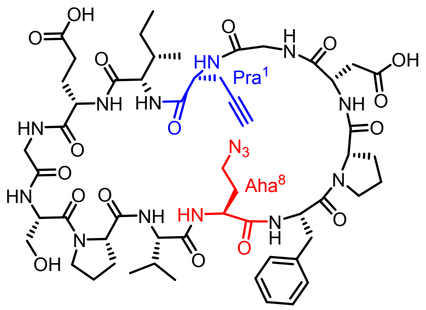

The linear peptide **3a** was synthesized by manual Fmoc-SPPS as described in **General Protocol 2** following which the N-terminal Fmoc group was removed with 20% piperidine + 5% formic acid in DMF (*v/v/v*, 2 x 5 min). The final peptide (0.05 mmol) with Pra and Aha incorporated at positions 1 and 8 respectively, was liberated from the resin with of a solution of 1% TFA in CH_2Cl_2 over 2 h. Following the removal of excess solvent the protected peptidyl product was lyophilized. The protected peptidyl product underwent head-to-tail cyclization in DMF initiated by HATU (76 mg, 0.2 mmol, 4 equiv.) and DIPEA (70 µL, 0.4 mmol, 8 equiv.) with reaction completion monitored by RP-HPLC. The coupling reagents were removed by diluting the solution in 1:1 CH_2Cl_2 prior to partitioning with three volumes of H_2O. The organic phase was collected and excess solvent removed *in vacuo*. The protecting groups were removed under the conditions described in **General Protocol 7** to afford the crude peptide, **3a** (41.5 mg, 74% yield [based on initial resin loading]) at approximately 40% purity.

Crude peptide **3a** (41.5 mg), was solubilized in a solution of 0.1% TFA in MeCN:MQ H_2O (2:8; *v/v*) at a concentration of ~20 mg/mL and purified by preparative RP-HPLC (1 x 2000 µL injection) employing a gradient of 15% – 60%B over 45 min (*ca.* 1%B/min) at a flow rate of 10 mL/min. Fractions were analyzed by RP-HPLC and LCMS for compound identification and lyophilized to afford the compound, **3a**, as a white amorphous powder (2.1 mg, 5% recovery [based on crude yield], 95% purity, 1% overall yield).

LCMS (ESI+): Mass calculated for [$C_{55}H_{75}N_{15}O_{17}$ + H] 1220.31; deconvoluted mass observed 1220.09 ± 0.11. Charge states; 611.08 $[M+2H]^{2+}$, 1221.01 $[M+H]^{+}$.

RP-HPLC: linear gradient = 5% – 95%B over 60 min, flow rate = 1.0 mL/min, l = 214 nm; t_R = 25.3 min.

Synthesis of **3b**

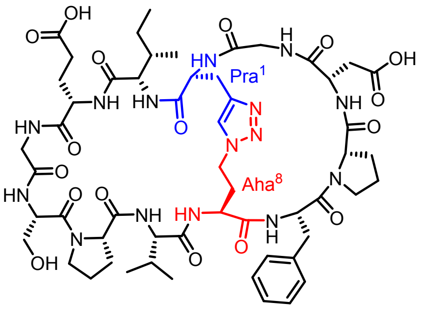

The linear peptide **3b** was synthesized by manual Fmoc-SPPS as described in **General Protocol 2.** 1,4-Triazole formation was facilitated under **General Protocol 3**, following which the N-terminal Fmoc group was removed with 20% piperidine + 5% formic acid in DMF (*v/v/v*, 2 x 5 min). The final peptide (0.05 mmol) with Pra and Aha incorporated at positions 1 and 8 respectively, was liberated from the resin with of a solution of 1% TFA in CH_2Cl_2 over 2 h. Following the removal of excess solvent the protected peptidyl product was lyophilized. The protected peptidyl product underwent head-to-tail cyclization in DMF initiated by HATU (76 mg, 0.2 mmol, 4 equiv.) and DIPEA (70 µL, 0.4 mmol, 8 equiv.) with reaction completion monitored by RP-HPLC. The coupling reagents were removed by diluting the solution in 1:1 CH_2Cl_2 prior to partitioning with three volumes of H_2O. The organic phase was collected and excess solvent removed *in vacuo*. The protecting groups were removed under the conditions described in **General Protocol 7** to afford the crude cyclized peptide, **3b** (25.4 mg, 42% yield [based on initial resin loading]) at approximately 60% purity.

Crude peptide **3b** (25.4 mg), was solubilized in a solution of 0.1% TFA in MeCN:MQ H_2O (2:8; *v/v*) at a concentration of ~25 mg/mL and purified by preparative RP-HPLC (1 x 1000 µL injection) employing a gradient of 15% – 60%B over 45 min (*ca.* 1%B/min) at a flow rate of 10 mL/min. Fractions were analyzed by RP-HPLC and LCMS for compound identification and lyophilized to afford the compound, **3b**, as a white amorphous powder (1.6 mg, 5% recovery [based on crude yield], 98% purity, 1% overall yield).

LCMS (ESI+): Mass calculated for [$C_{55}H_{75}N_{15}O_{17}$ + H] 1220.31; deconvoluted mass observed 1219.97 ± 0.26. Charge states; 610.89 $[M+2H]^{2+}$, 1221.15 $[M+H]^{+}$.

RP-HPLC: linear gradient = 5% – 95%B over 60 min, flow rate = 1.0 mL/min, l = 214 nm; t_R = 23.7 min.

Synthesis of **3c**

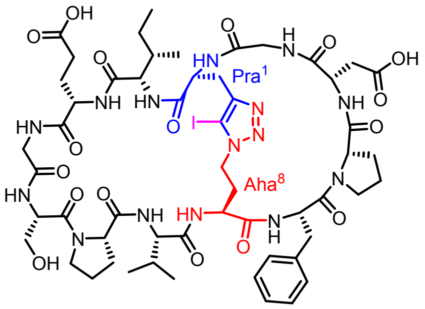

The linear peptide **3c** was synthesized by manual Fmoc-SPPS as described in **General Protocol 2.** 5-Iodo-1,4-triazole formation was facilitated under **General Protocol 4**, following which the N-terminal Fmoc group was removed with 20% piperidine + 5% formic acid in DMF (2 x 5 min). The final peptide (0.05 mmol) with Pra and Aha incorporated at positions 1 and 8 respectively, was liberated from the resin with of a solution of 1% TFA in CH_2Cl_2 over 2 h. Following the removal of excess solvent the protected peptidyl product was lyophilized. The protected peptidyl product underwent head-to-tail cyclization in DMF initiated by HATU (76 mg, 0.2 mmol, 4 equiv.) and DIPEA (70 µL, 0.4 mmol, 8 equiv.) with reaction completion monitored by RP-HPLC. The coupling reagents were removed by diluting the solution in 1:1 CH_2Cl_2 prior to partitioning with three volumes of H_2O. The organic phase was collected and excess solvent removed *in vacuo*. The protecting groups were removed under the conditions described in **General Protocol 7** to afford the crude cyclized peptide, **3c** (29.9 mg, 44% yield [based on initial resin loading]) at approximately 55% purity.

Crude peptide **3c** (44 mg), was solubilized in a solution of 0.1% TFA in MeCN:MQ H_2O (2:8; *v/v*) at a concentration of ~20 mg/mL and purified by preparative RP-HPLC (1 x 2000 µL injection) employing a gradient of 15% – 60%B over 45 min (*ca.* 1%B/min) at a flow rate of 10 mL/min. Fractions were analyzed by RP-HPLC and LCMS for compound identification and lyophilized to afford the compound, **3c**, as a white amorphous powder (0.5 mg, 2% recovery [based on crude yield], 99% purity, 1% overall yield).

LCMS (ESI+): Mass calculated for $[C_{55}H_{74}IN_{15}O_{17} + H]$ 1346.21; deconvoluted mass observed 1345.55 ± 0.06. Charge states; 673.75 $[M+2H]^{2+}$, 1346.59 $[M+H]^{+}$.

RP-HPLC: linear gradient = 5% – 95%B over 60 min, flow rate = 1.0 mL/min, l = 214 nm; t_R = 25.1 min.

Synthesis of **3d**

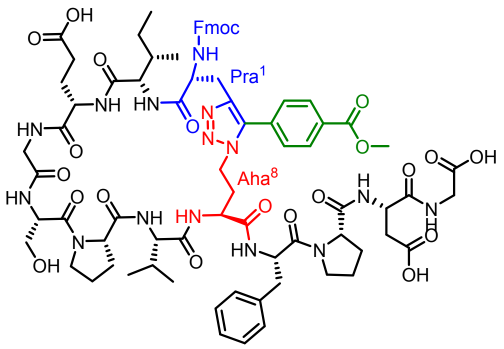

The linear peptide **3d** was synthesized by manual Fmoc-SPPS as described in **General Protocol 2.** 5-Iodo-1,4-triazole formation was facilitated under **General Protocol 4**. Suzuki-Miyaura coupling was mediated by **General Protocol 5**. The final cyclized peptide was liberated from the resin and its protecting groups simultaneously removed under the conditions described in **General Protocol 7** to afford the crude cyclized peptide, **3d** (11.5 mg, 15% yield [based on initial resin loading]) at approximately 30% purity.

Crude peptide **3d** (11.5 mg), was solubilized in a solution of 0.1% TFA in MeCN:MQ H_2O (3:7; *v/v*) at a concentration of ~5 mg/mL and purified by preparative RP-HPLC (2 x 1000 µL injection) employing a gradient of 30% – 75%B over 45 min (*ca.* 1%B/min) at a flow rate of 10 mL/min. Fractions were analyzed by RP-HPLC and LCMS for compound identification and lyophilized to afford the compound, **3d**, as a white amorphous powder (0.7 mg, 6% recovery [based on crude yield], 96% purity, 1% overall yield).

LCMS (ESI+): Mass calculated for $[C_{78}H_{95}N_{15}O_{22} + H]$ 1594.70; deconvoluted mass observed 1594.47 ± 0.10. Charge states; 798.20 $[M+2H]^{2+}$, 1595.54 $[M+H]^{+}$.

RP-HPLC: linear gradient = 5% – 95%B over 60 min, flow rate = 1.0 mL/min, l = 214 nm; t_R = 33.4 min.

Synthesis of **3e**

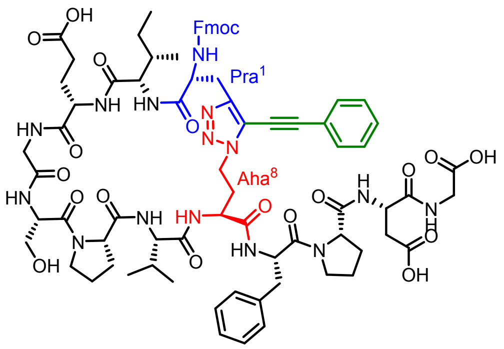

The linear peptide **3e** was synthesized by manual Fmoc-SPPS as described in **General Protocol 2.** 5-Iodo-1,4-triazole formation was facilitated under **General Protocol 4**. Sonogashira coupling was mediated by **General Protocol 6**. The final cyclized peptide was liberated from the resin and its protecting groups simultaneously removed under the conditions described in **General Protocol 7** to afford the crude cyclized peptide, **3e** (2 mg, 3% yield [based on initial resin loading]) at approximately 35% purity.

Crude peptide **3e** (2 mg), was solubilized in a solution of 0.1% TFA in MeCN:MQ H_2O (3:7; *v/v*) at a concentration of ~1 mg/mL and purified by semi-preparative RP-HPLC (2 x 1000 µL injection) employing a gradient of 30% – 75%B over 45 min (*ca.* 1%B/min) at a flow rate of 10 mL/min. Fractions were analyzed by RP-HPLC and LCMS for compound identification and lyophilized to afford the compound, **3e**, as a white amorphous powder (0.4 mg, 20% recovery [based on crude yield], 95% purity, 1% overall yield).

LCMS (ESI+): Mass calculated for [$C_{78}H_{93}N_{15}O_{20}$ + H] 1560.69; deconvoluted mass observed 1560.55 ± 0.02. Charge states; 781.28 $[M+2H]^{2+}$, 1561.53 $[M+H]^{+}$.

RP-HPLC: linear gradient = 5% – 95%B over 60 min, flow rate = 1.0 mL/min, l = 214 nm; t_R = 34.5 min.

Synthesis of **4a**

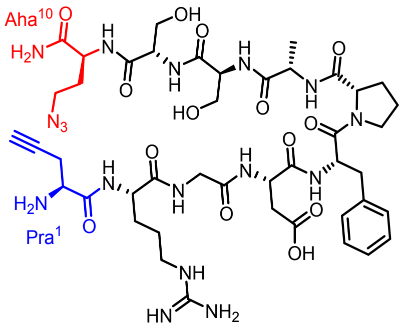

The linear peptide **4a** was synthesized by automated Fmoc-SPPS as described in **General Protocol 1**. The final peptide (0.05 mmol) with Pra and Aha incorporated at positions 1 and 10 respectively was liberated from the resin and its protecting groups simultaneously removed under the conditions described in **General Protocol 7** to afford the crude peptide, **4a** (27.5 mg, 52% yield [based on initial resin loading]) at approximately 88% purity.

Crude peptide **4a** (27.5 mg), was solubilized in a solution of 0.1% TFA in MeCN:MQ H_2O (2:8; *v/v*) at a concentration of ~20 mg/mL and purified by preparative RP-HPLC (1 x 1300 µL injection) employing a gradient of 15% – 60%B over 45 min (*ca.* 1%B/min) at a flow rate of 10 mL/min. Fractions were analyzed by RP-HPLC and LCMS for compound identification and lyophilized to afford the compound, **4a**, as a white amorphous powder (6.1 mg, 22% recovery [based on crude yield], 99% purity, 12% overall yield).

LCMS (ESI+): Mass calculated for [$C_{44}H_{65}N_{17}O_{14}$ + H] 1056.11; deconvoluted mass observed 1056.11 ± 0.18. Charge states; 529.12 $[M+2H]^{2+}$, 1056.98 $[M+H]^{+}$.

RP-HPLC: linear gradient = 5% – 95%B over 60 min, flow rate = 1.0 mL/min, l = 214 nm; t_R = 17.7 min.

Synthesis of **4b**

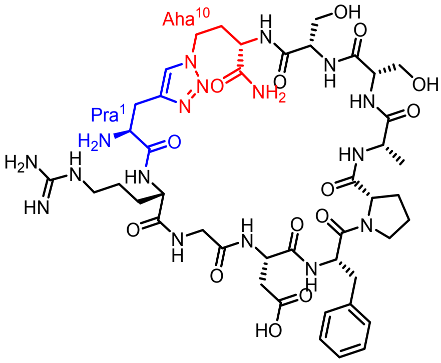

The linear precursor for peptide **4b** was synthesized by automated Fmoc-SPPS as described in **General Protocol 1** with Pra and Aha incorporated at positions 1 and 10 respectively. Following synthesis of the linear peptide, the peptidyl resin (0.05 mmol) was subjected to disubstituted copper-catalyzed azide-alkyne cycloaddition (CuAAC) conditions, as described in **General Protocol 3**. The final cyclized peptide was liberated from the resin and its protecting groups simultaneously removed under the conditions described in **General Protocol 7** to afford the crude cyclized peptide, **4b** (12.6 mg, 24% yield [based on initial resin loading]) at approximately 89% purity.

Crude peptide **4b** (24 mg), was solubilized in a solution of 0.1% TFA in MeCN:MQ H_2O (2:8; *v/v*) at a concentration of ~20 mg/mL and purified by preparative RP-HPLC (1 x 1200 µL injection) employing a gradient of 15% – 60%B over 45 min (*ca.* 1%B/min) at a flow rate of 10 mL/min. Fractions were analyzed by RP-HPLC and LCMS for compound identification and lyophilized to afford the compound, **4b**, as a white amorphous powder (4.4 mg, 35% recovery [based on crude yield], 99% purity, 8% overall yield).

LCMS (ESI+): Mass calculated for [$C_{44}H_{65}N_{17}O_{14}$ + H] 1056.11; deconvoluted mass observed 1056.07 ± 0.22. Charge states; 529.11 $[M+2H]^{2+}$, 1056.91 $[M+H]^{+}$.

RP-HPLC: linear gradient = 5% – 95%B over 60 min, flow rate = 1.0 mL/min, l = 214 nm; t_R = 16.4 min.

Synthesis of **4c**

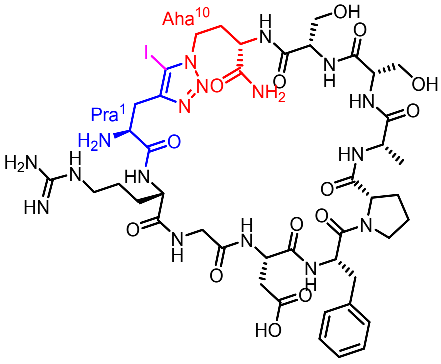

The linear precursor for peptide **4c** was synthesized by automated Fmoc-SPPS as described in **General Protocol 1** with Pra and Aha incorporated at positions 1 and 10 respectively. Following synthesis of the linear peptide, the peptidyl resin (0.05 mmol) was subjected to trisubstituted copper-catalyzed azide-alkyne cycloaddition (CuAAC) conditions, as described in **General Protocol 4**. The final cyclized peptide was liberated from the resin and its protecting groups simultaneously removed under the conditions described in **General Protocol 7** to afford the crude cyclized peptide, **4c** (18.8 mg, 32% yield [based on initial resin loading]) at approximately 81% purity.

Crude peptide **4c** (18.8 mg), was solubilized in a solution of 0.1% TFA in MeCN:MQ H_2O (2:8; *v/v*) at a concentration of ~20 mg/mL and purified by preparative RP-HPLC (1 x 1000 µL injection) employing a gradient of 15% – 60%B over 45 min (*ca.* 1%B/min) at a flow rate of 10 mL/min. Fractions were analyzed by RP-HPLC and LCMS for compound identification and lyophilized to afford the compound, **4c**, as a white amorphous powder (0.6 mg, 3% recovery [based on crude yield], 98% purity, 1% overall yield).

LCMS (ESI+): Mass calculated for [$C_{44}H_{64}IN_{17}O_{14}$ + H] 1182.01; deconvoluted mass observed 1181.85 ± 0.36. Charge states; 592.05 $[M+2H]^{2+}$, 1182.59 $[M+H]^{+}$.

RP-HPLC: linear gradient = 5% – 95%B over 60 min, flow rate = 1.0 mL/min, l = 214 nm; t_R = 17.0 min.

Synthesis of **4d**

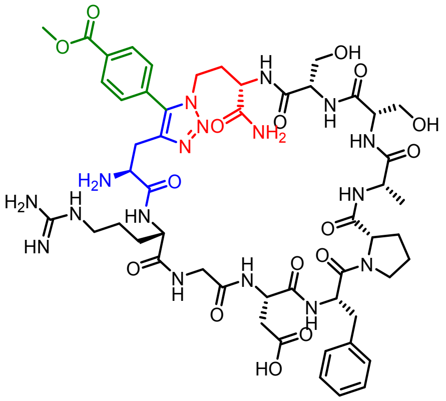

The linear precursor for peptide **4d** was synthesized by automated Fmoc-SPPS as described in **General Protocol 1** with Pra and Aha incorporated at positions 1 and 10 respectively. Following synthesis of the linear peptide, the peptidyl resin (0.05 mmol) was subjected to trisubstituted copper-catalyzed azide-alkyne cycloaddition (CuAAC) conditions, as described in **General Protocol 4**. The cyclized peptide underwent an on-resin Suzuki coupling with 4-methoxycarbonylphenylboronic acid pinacol ester as described by **General Method 5**. The final cyclized peptide was liberated from the resin and its protecting groups simultaneously removed under the conditions described in **General Protocol 7** to afford the crude cyclized peptide, **4d** (20.2 mg, 34% yield [based on initial resin loading]) at approximately 70% purity.

Crude peptide **4d** (20.2 mg), was solubilized in a solution of 0.1% TFA in MeCN:MQ H_2O (2:8; *v/v*) at a concentration of ~10 mg/mL and purified by preparative RP-HPLC (1 x 2000 μL injection) employing a gradient of 15% – 60%B over 45 min (*ca.* 1%B/min) at a flow rate of 10 mL/min. Fractions were analyzed by RP-HPLC and LCMS for compound identification and lyophilized to afford the compound, **4d**, as a white amorphous powder (0.5 mg, 2% recovery [based on crude yield], 99% purity, 1% overall yield).

LCMS (ESI+): Mass calculated for [$C_{52}H_{71}N_{17}O_{16}$ + H] 1190.24; deconvoluted mass observed 1189.91 ± 0.64. Charge states; 596.18 $[M+2H]^{2+}$, 1190.50 $[M+H]^{+}$.

RP-HPLC: linear gradient = 5% – 95%B over 60 min, flow rate = 1.0 mL/min, l = 214 nm; t_R = 18.6 min.

Synthesis of **4e**

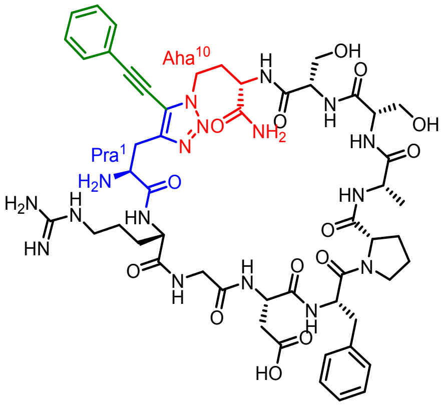

The linear precursor for peptide **4e** was synthesized by automated Fmoc-SPPS as described in **General Protocol 1** with Pra and Aha incorporated at positions 1 and 10 respectively. Following synthesis of the linear peptide, the peptidyl resin (0.05 mmol) was subjected to trisubstituted copper-catalyzed azide-alkyne cycloaddition (CuAAC) conditions, as described in **General Protocol 4**. The cyclized peptide underwent an on-resin Sonogashira coupling with phenylacetylene as described by **General Method 6**. The final cyclized peptide was liberated from the resin and its protecting groups simultaneously removed under the conditions described in **General Protocol 7** to afford the crude cyclized peptide, **4e** (17.1 mg, 30% yield [based on initial resin loading]) at approximately 60% purity.

Crude peptide **4e** (17.1 mg), was solubilized in a solution of 0.1% TFA in MeCN:MQ H_2O (2:8; *v/v*) at a concentration of ~8.5 mg/mL and purified by preparative RP-HPLC (2 x 1000 µL injection) employing a gradient of 15% – 60%B over 45 min (*ca.* 1%B/min) at a flow rate of 10 mL/min. Fractions were analyzed by RP-HPLC and LCMS for compound identification and lyophilized to afford the compound, **4e**, as a white amorphous powder (1.02 mg, 3% recovery [based on crude yield], 98% purity, 2% overall yield).

LCMS (ESI+): Mass calculated for [$C_{52}H_{69}N_{17}O_{14}$ + H] 1156.23; deconvoluted mass observed 1155.95 ± 0.47. Charge states; 579.14 $[M+2H]^{2+}$, 1156.61 $[M+H]^{+}$.

RP-HPLC: linear gradient = 5% – 95%B over 60 min, flow rate = 1.0 mL/min, l = 214 nm; t_R = 20.6 min.

Synthesis of **5a**

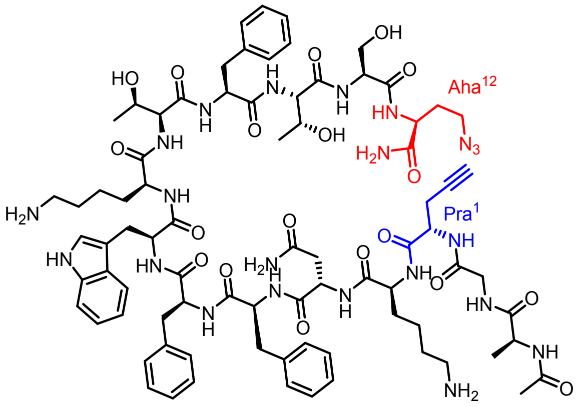

The linear peptide **5a** was synthesized by automated Fmoc-SPPS as described in **General Protocol 1**. The final peptide (0.05 mmol) with Pra and Aha incorporated at positions 1 and 12 respectively was liberated from the resin and its protecting groups simultaneously removed under the conditions described in **General Protocol 7** to afford the crude peptide, **5a** (55.6 mg, 66% yield [based on initial resin loading]) at approximately 65% purity.

Crude peptide **5a** (55.6 mg), was solubilized in a solution of 0.1% TFA in MeCN:MQ H_2O (2:8; *v/v*) at a concentration of ~25 mg/mL and purified by preparative RP-HPLC (1 x 2000 µL injection) employing a gradient of 15% – 60%B over 45 min (*ca.* 1%B/min) at a flow rate of 10 mL/min. Fractions were analyzed by RP-HPLC and LCMS for compound identification and lyophilized to afford the compound, **5a**, as a white amorphous powder (6.8 mg, 12% recovery [based on crude yield], 98% purity, 8% overall yield).

LCMS (ESI+): Mass calculated for [$C_{81}H_{110}N_{22}O_{19}$ + H] 1695.91; deconvoluted mass observed 1695.32 ± 0.80. Charge states; 848.94 $[M+2H]^{2+}$, 1695.75 $[M+H]^{+}$.

RP-HPLC: linear gradient = 5% – 95%B over 60 min, flow rate = 1.0 mL/min, l = 214 nm; t_R = 27.4 min.

Synthesis of **5b**

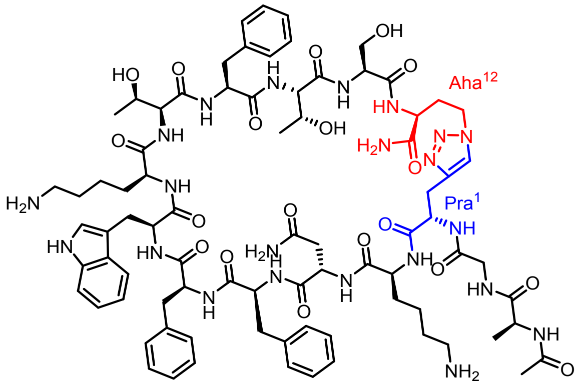

The linear precursor for peptide **5b** was synthesized by automated Fmoc-SPPS as described in **General Protocol 1** with Pra and Aha incorporated at positions 1 and 12 respectively. Following synthesis of the linear peptide, the peptidyl resin (0.05 mmol) was subjected to disubstituted copper-catalyzed azide-alkyne cycloaddition (CuAAC) conditions, as described in **General Protocol 3 (modified solvent system of neat DMSO)**. The final cyclized peptide was liberated from the resin and its protecting groups simultaneously removed under the conditions described in **General Protocol 7** to afford the crude cyclized peptide, **5b** (3.86 mg, 4% yield [based on initial resin loading]) at approximately 75% purity.

Crude peptide **5b** (3.86 mg), was solubilized in a solution of 0.1% TFA in MeCN:MQ H_2O (2:8; *v/v*) at a concentration of ~3 mg/mL and purified by preparative RP-HPLC (1 x 1000 µL injection) employing a gradient of 15% – 60%B over 45 min (*ca.* 1%B/min) at a flow rate of 10 mL/min. Fractions were analyzed by RP-HPLC and LCMS for compound identification and lyophilized to afford the compound, **5b**, as a white amorphous powder (0.7 mg, 18% recovery [based on crude yield], 98% purity, 1% overall yield).

LCMS (ESI+): Mass calculated for $[C_{81}H_{110}N_{22}O_{19} + H]$ 1695.91; deconvoluted mass observed 1695.71 ± 0.21. Charge states; 848.93 $[M+2H]^{2+}$, 1696.56 $[M+H]^{+}$.

RP-HPLC: linear gradient = 5% – 95%B over 60 min, flow rate = 1.0 mL/min, l = 214 nm; t_R = 25.5 min.

Synthesis of **5c**

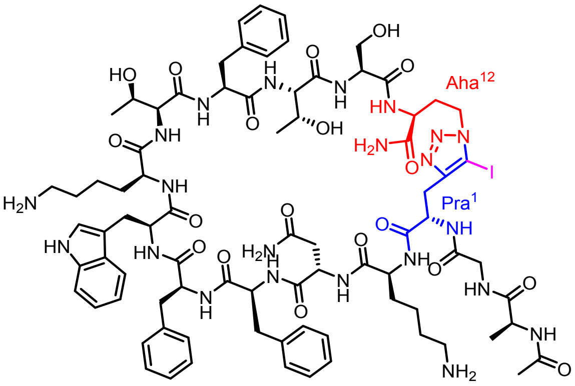

The linear precursor for peptide **5c** was synthesized by automated Fmoc-SPPS as described in **General Protocol 1** with Pra and Aha incorporated at positions 1 and 12 respectively. Following synthesis of the linear peptide, the peptidyl resin (0.05 mmol) was subjected to trisubstituted copper-catalyzed azide-alkyne cycloaddition (CuAAC) conditions, as described in **General Protocol 4 (modified solvent system of DMSO:DMF [1:1, *v/v*])**. The final cyclized peptide was liberated from the resin and its protecting groups simultaneously removed under the conditions described in **General Protocol 7** to afford the crude cyclized peptide, **5c** (5 mg, 5% yield [based on initial resin loading]) at approximately 45% purity.

Crude peptide **5c** (5 mg), was solubilized in a solution of 0.1% TFA in MeCN:MQ H_2O (2:8; *v/v*) at a concentration of ~5 mg/mL and purified by preparative RP-HPLC (1 x 1000 µL injection) employing a gradient of 15% – 60%B over 45 min (*ca.* 1%B/min) at a flow rate of 10 mL/min. Fractions were analyzed by RP-HPLC and LCMS for compound identification and lyophilized to afford the compound, **5c**, as a white amorphous powder (0.5 mg, 10% recovery [based on crude yield], 95% purity, 1% overall yield).

LCMS (ESI+): Mass calculated for [$C_{81}H_{109}IN_{22}O_{19}$ + H] 1821.80; deconvoluted mass observed 1821.69 ± 0.22. Charge states; 911.92 $[M+2H]^{2+}$, 1822.53 $[M+H]^{+}$.

RP-HPLC: linear gradient = 5% – 95%B over 60 min, flow rate = 1.0 mL/min, l = 214 nm; t_R = 26.0 min.

Synthesis of **5d**

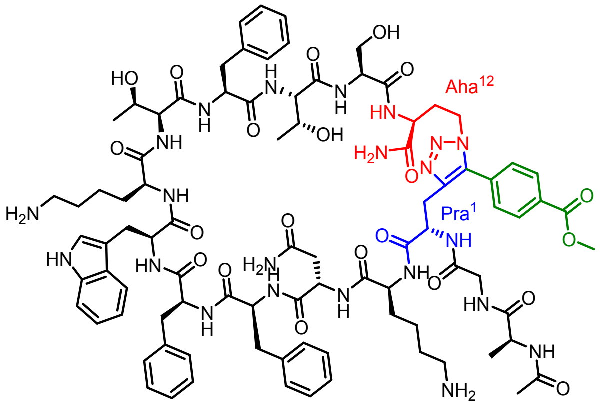

The linear precursor for peptide **5d** was synthesized by automated Fmoc-SPPS as described in **General Protocol 1** with Pra and Aha incorporated at positions 1 and 12 respectively. Following synthesis of the linear peptide, the peptidyl resin (0.05 mmol) was subjected to trisubstituted copper-catalyzed azide-alkyne cycloaddition (CuAAC) conditions, as described in **General Protocol 4**. The cyclized peptide underwent an on-resin Suzuki coupling with 4-methoxycarbonylphenylboronic acid pinacol ester as described by **General Method 5**. The final cyclized peptide was liberated from the resin and its protecting groups simultaneously removed under the conditions described in **General Protocol 7** to afford the crude cyclized peptide, **5d** (4 mg, 5% yield [based on initial resin loading]) at approximately 55% purity.

Crude peptide **5d** (4 mg), was solubilized in a solution of 0.1% TFA in MeCN:MQ H_2O (2:8; *v/v*) at a concentration of ~4 mg/mL and purified by preparative RP-HPLC (1 x 1000 µL injection) employing a gradient of 15% – 60%B over 45 min (*ca.* 1%B/min) at a flow rate of 10 mL/min. Fractions were analyzed by RP-HPLC and LCMS for compound identification and lyophilized to afford the compound, **5d**, as a white amorphous powder (0.5 mg, 13% recovery [based on crude yield], 96% purity, 1% overall yield).

LCMS (ESI+): Mass calculated for [$C_{89}H_{116}N_{22}O_{21}$ + H] 1830.04; deconvoluted mass observed 1830.06. Charge states; 915.03 $[M+2H]^{2+}$.

RP-HPLC: linear gradient = 5% – 95%B over 60 min, flow rate = 1.0 mL/min, l = 214 nm; t_R = 26.4 min.

Synthesis of **5e**

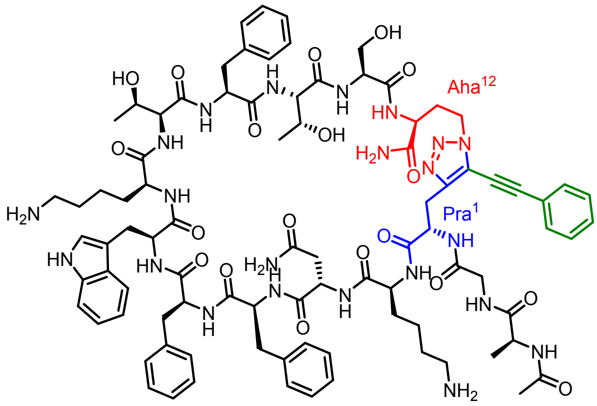

The linear precursor for peptide **5e** was synthesized by automated Fmoc-SPPS as described in **General Protocol 1** with Pra and Aha incorporated at positions 1 and 12 respectively. Following synthesis of the linear peptide, the peptidyl resin (0.05 mmol) was subjected to trisubstituted copper-catalyzed azide-alkyne cycloaddition (CuAAC) conditions, as described in **General Protocol 4**. The cyclized peptide underwent an on-resin Sonogashira coupling with phenylacetylene as described by **General Method 6**. The final cyclized peptide was liberated from the resin and its protecting groups simultaneously removed under the conditions described in **General Protocol 7** to afford the crude cyclized peptide, **5e** (5 mg, 5% yield [based on initial resin loading]) at approximately 50% purity.

Crude peptide **5e** (5 mg), was solubilized in a solution of 0.1% TFA in MeCN:MQ H_2O (2:8; *v/v*) at a concentration of ~5 mg/mL and purified by preparative RP-HPLC (1 x 1000 µL injection) employing a gradient of 15% – 60%B over 45 min (*ca.* 1%B/min) at a flow rate of 10 mL/min. Fractions were analyzed by RP-HPLC and LCMS for compound identification and lyophilized to afford the compound, **5e**, as a white amorphous powder (0.5 mg, 10% recovery [based on crude yield], 95% purity, 1% overall yield).

LCMS (ESI+): Mass calculated for [$C_{89}H_{114}N_{22}O_{19}$ + H] 1796.03; deconvoluted mass observed 1796.03. Charge states; 898.97 $[M+2H]^{2+}$.

RP-HPLC: linear gradient = 5% – 95%B over 60 min, flow rate = 1.0 mL/min, l = 214 nm; t_R = 27.2 min.

Characterisation

Reverse Phase – High Performance Liquid Chromatography (RP-HPLC) and Liquid Chromatography Mass Spectrometry (LCMS)

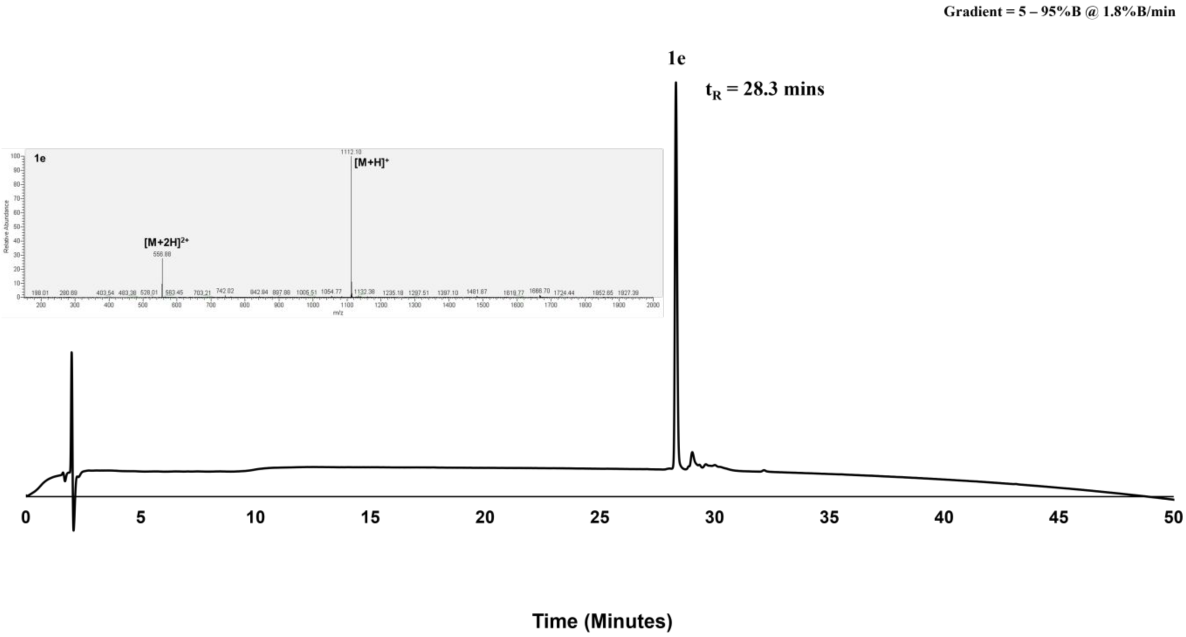

Supporting Information Figure S6. Analytical RP-HPLC chromatogram (214 nm) of purified peptide, **1e**, *ca.* 96% as analysed by peak area. **RP-HPLC:** linear gradient = 5% – 95%B over 60 min, flow rate = 1.0 mL/min, l = 214 nm; t_R = 28.3 mins. **LCMS (ESI+)** inset of **1e**, mass calculated for [$C_{54}H_{74}N_{14}O_{12}$ + H] 1111.27; deconvoluted mass observed: 1111.43 ± 0.45. Charge states; 556.88 $[M+2H]^{2+}$, 1112.10 $[M+H]^{+}$.

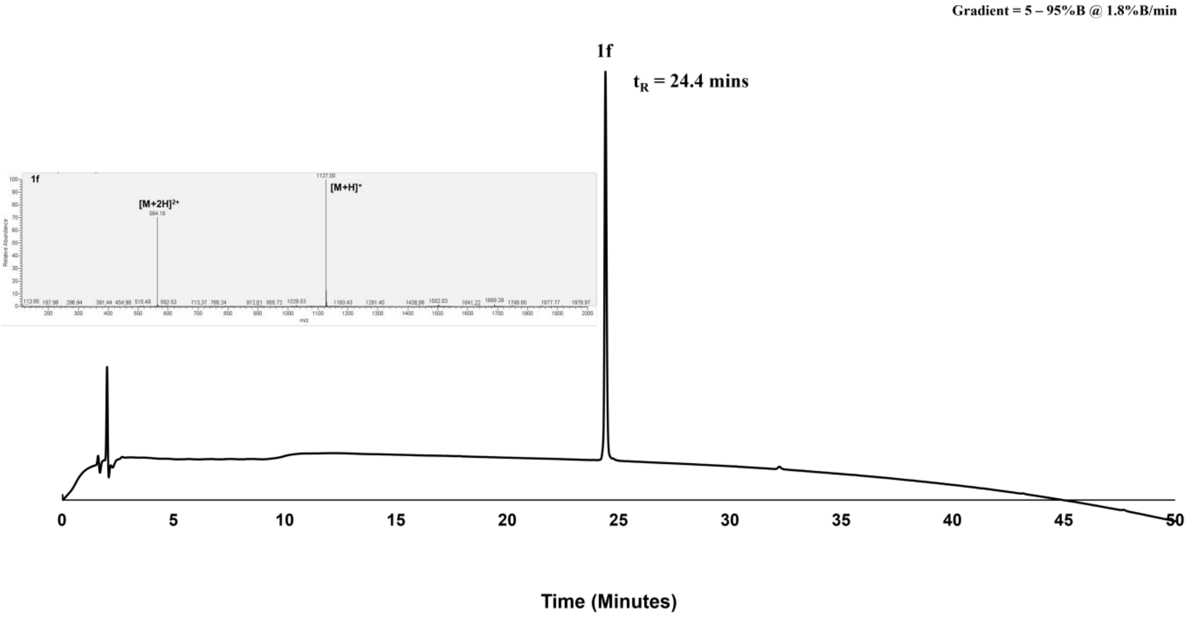

Supporting Information Figure S7. Analytical RP-HPLC chromatogram (214 nm) of purified peptide, **1f**, *ca.* 99% as analysed by peak area. **RP-HPLC:** linear gradient = 5% – 95%B over 60 min, flow rate = 1.0 mL/min, l = 214 nm; t_R = 24.4 mins. **LCMS (ESI+)** inset of **1f**, mass calculated for [$C_{54}H_{75}N_{15}O_{12}$ + H] 1126.29; deconvoluted mass observed: 1126.18 ± 0.25. Charge states; 564.18 $[M+2H]^{2+}$, 1127.00 $[M+H]^{+}$.

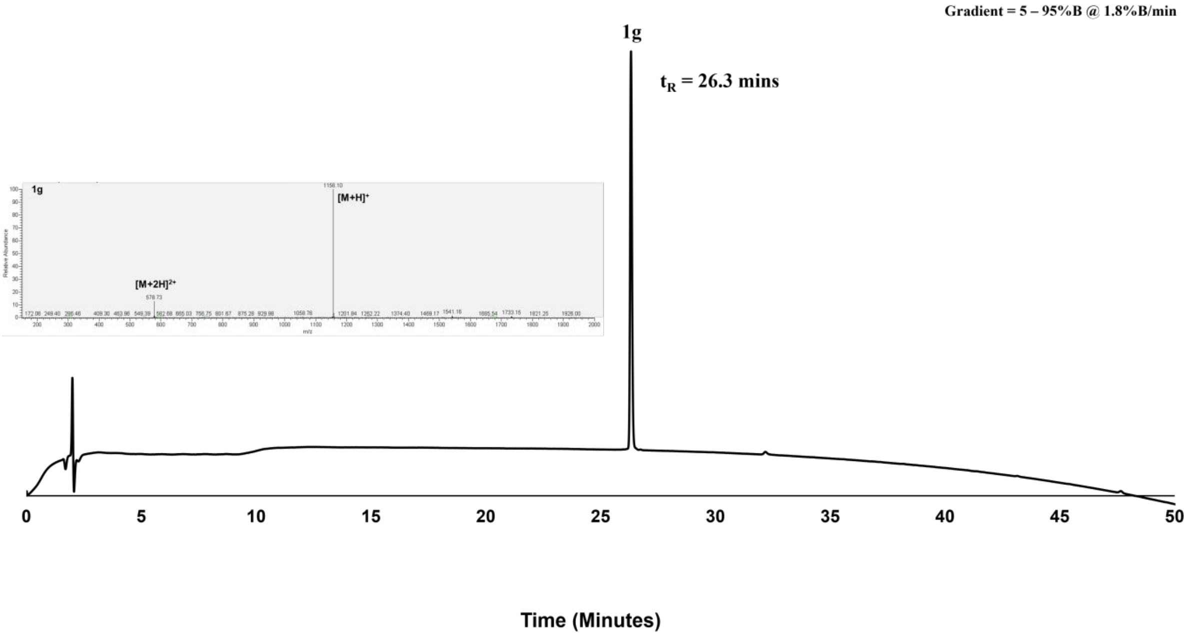

Supporting Information Figure S8. Analytical RP-HPLC chromatogram (214 nm) of purified peptide, **1g**, *ca.* 99% as analysed by peak area. **RP-HPLC:** linear gradient = 5% – 95%B over 60 min, flow rate = 1.0 mL/min, l = 214 nm; t_R = 26.3mins. **LCMS (ESI+)** inset of **1g**, mass calculated for [$C_{55}H_{74}N_{14}O_{14}$ + H] 1155.28; deconvoluted mass observed: 1155.28 ± 0.25. Charge states; 578.73 $[M+2H]^{2+}$, 1156.10 $[M+H]^{+}$.

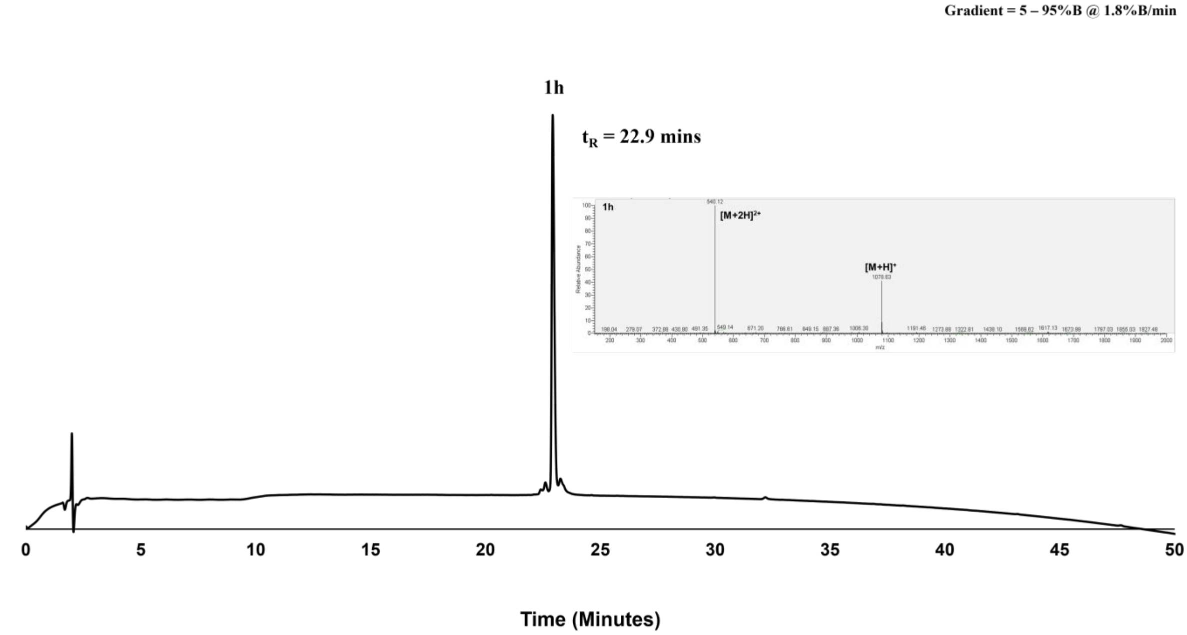

Supporting Information Figure S9. Analytical RP-HPLC chromatogram (214 nm) of purified peptide, **1h**, *ca.* 95% as analysed by peak area. **RP-HPLC:** linear gradient = 5% – 95%B over 60 min, flow rate = 1.0 mL/min, l = 214 nm; t_R = 22.9 mins. **LCMS (ESI+)** inset of **1h**, mass calculated for [$C_{50}H_{75}N_{15}O_{12}$ + H] 1078.24; deconvoluted mass observed: 1077.94 ± 0.43. Charge states; 540.12 $[M+2H]^{2+}$, 1078.63 $[M+H]^{+}$.

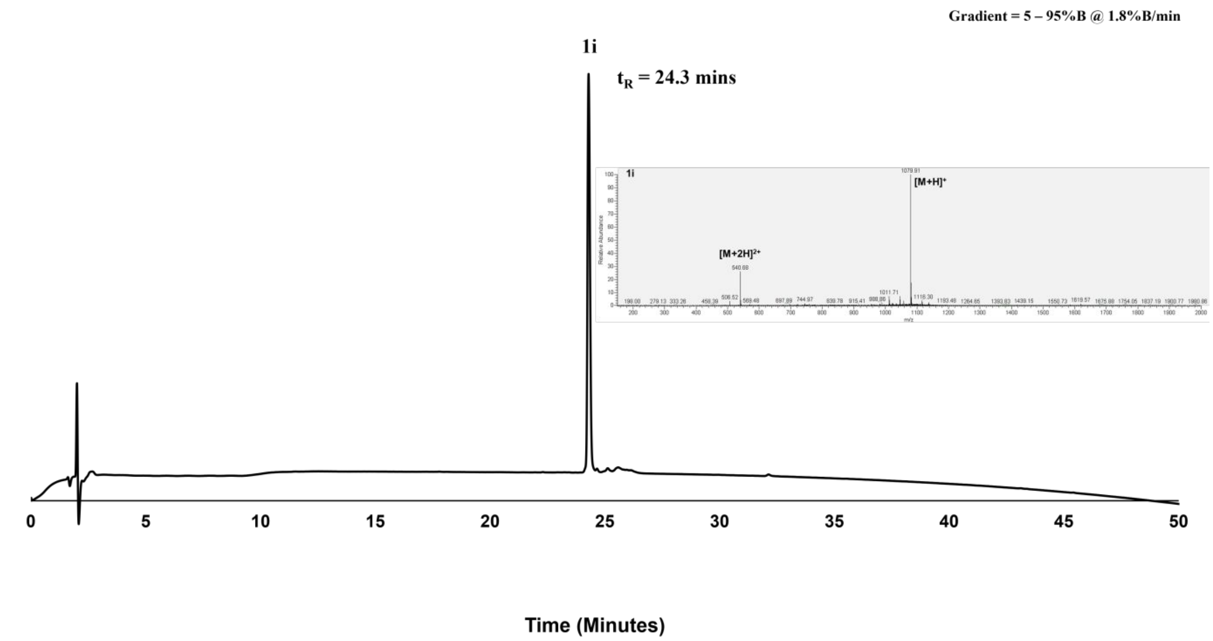

Supporting Information Figure S10. Analytical RP-HPLC chromatogram (214 nm) of purified peptide, **1i**, *ca.* 95% as analysed by peak area. **RP-HPLC:** linear gradient = 5% – 95%B over 60 min, flow rate = 1.0 mL/min, l = 214 nm; t_R = 24.3 mins. **LCMS (ESI+)** inset of **1i**, mass calculated for [$C_{54}H_{74}N_{14}O_{13}$ + H] 1079.24; deconvoluted mass observed: 1079.14 ± 0.31. Charge states; 540.69 $[M+2H]^{2+}$, 1079.91 $[M+H]^{+}$.

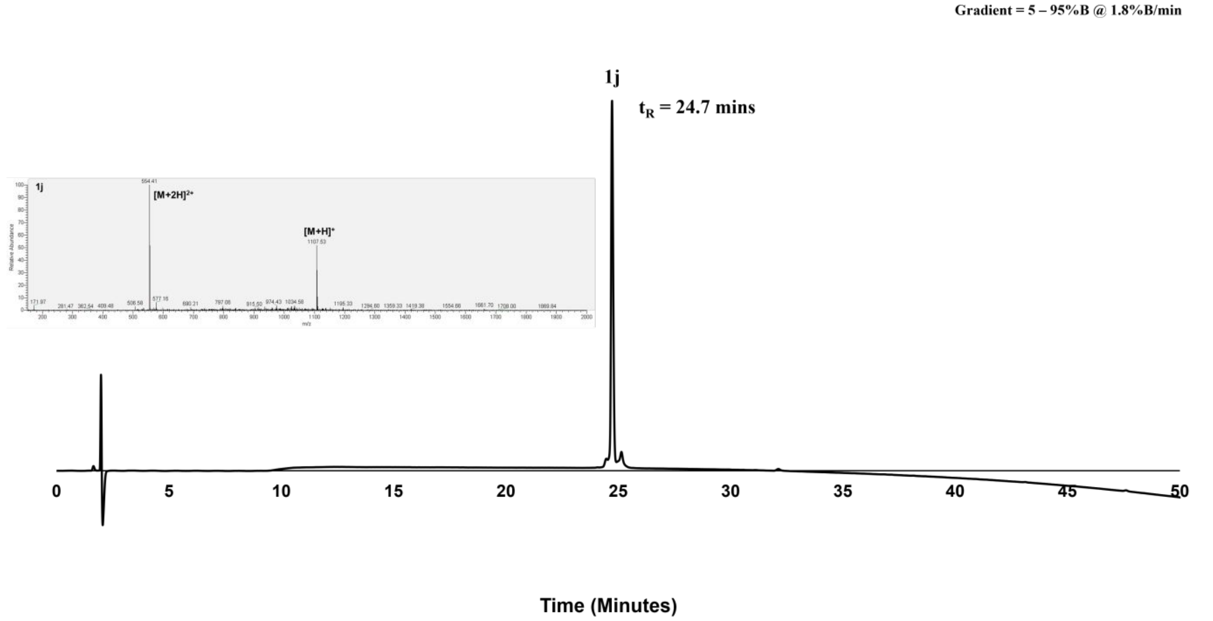

Supporting Information Figure S11. Analytical RP-HPLC chromatogram (214 nm) of purified peptide, **1j**, *ca.* 94% as analysed by peak area. **RP-HPLC:** linear gradient = 5% – 95%B over 60 min, flow rate = 1.0 mL/min, l = 214 nm; t_R = 24.7 mins. **LCMS (ESI+)** inset of **1j**, mass calculated for [$C_{51}H_{74}N_{14}O_{14}$ + H] 1107.24; deconvoluted mass observed: 1106.68 ± 0.21. Charge states; 554.41 $[M+2H]^{2+}$, 1107.53 $[M+H]^{+}$.

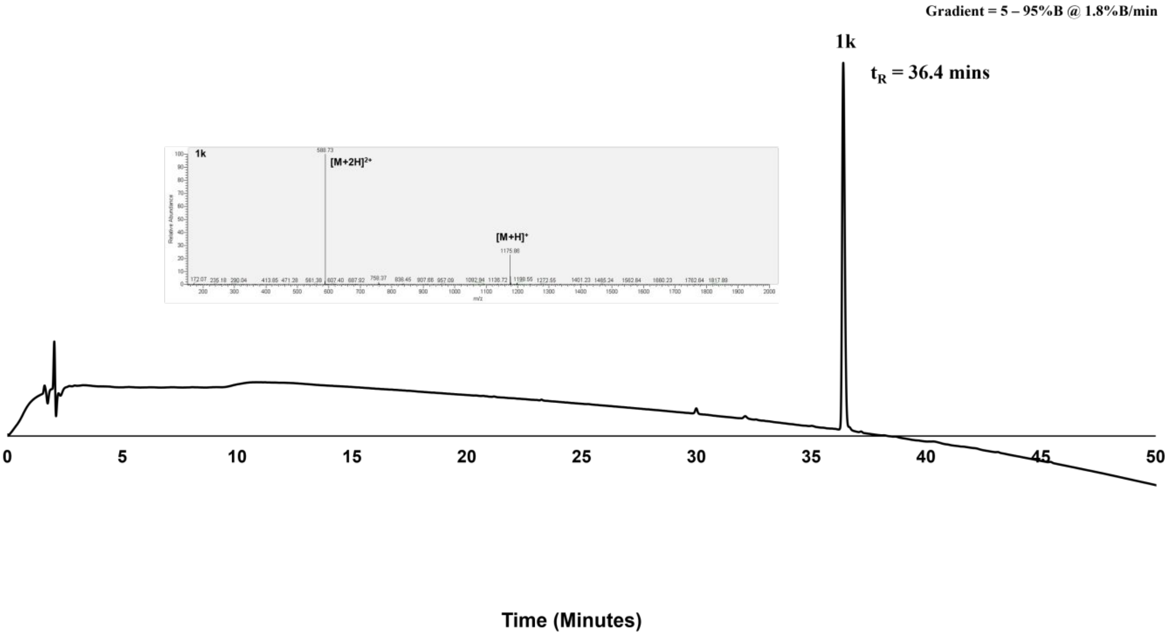

Supporting Information Figure S12. Analytical RP-HPLC chromatogram (214 nm) of purified peptide, **1k**, *ca.* 98% as analysed by peak area. **RP-HPLC:** linear gradient = 5% – 95%B over 60 min, flow rate = 1.0 mL/min, l = 214 nm; t_R = 36.4 mins. **LCMS (ESI+)** inset of **1k**, mass calculated for [$C_{58}H_{90}N_{14}O_{12}$ + H] 1175.44; deconvoluted mass observed: 1175.16 ± 0.42. Charge states; 588.73 $[M+2H]^{2+}$, 1175.86 $[M+H]^{+}$.

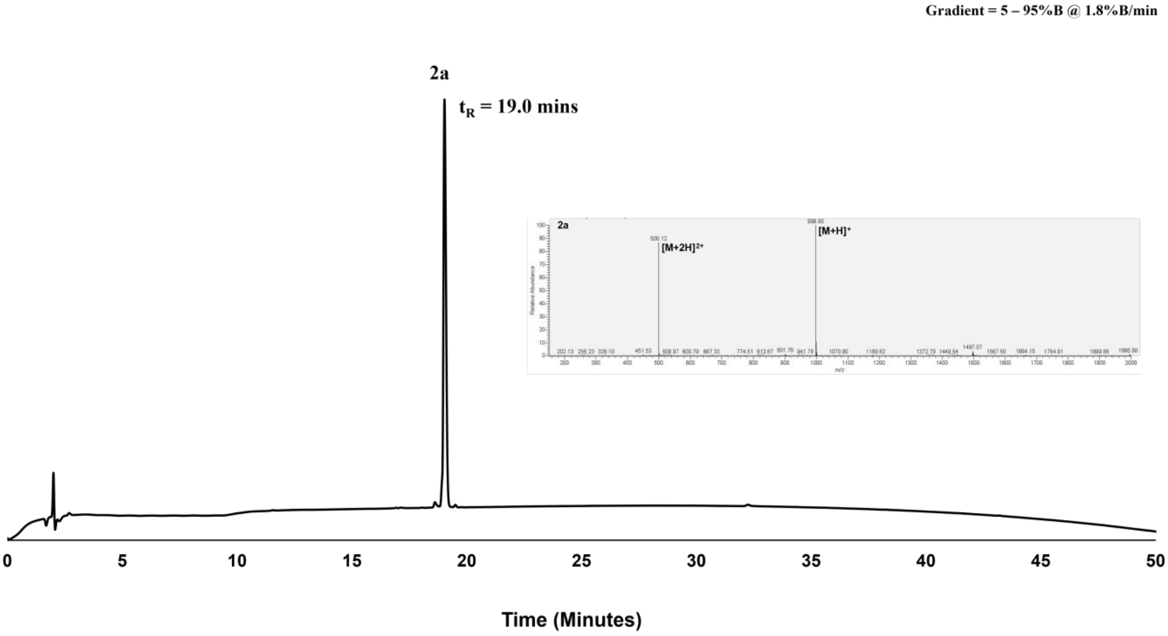

Supporting Information Figure S13. Analytical RP-HPLC chromatogram (214 nm) of purified peptide, **2a**, *ca.* 97% as analysed by peak area. **RP-HPLC:** linear gradient = 5% – 95%B over 60 min, flow rate = 1.0 mL/min, l = 214 nm; t_R = 19.0 mins. **LCMS (ESI+)** inset of **2a**, mass calculated for [$C_{43}H_{67}N_{17}O_{11}$ + H] 998.12; deconvoluted mass observed: 998.10 ± 0.21. Charge states; 500.12 $[M+2H]^{2+}$, 998.95 $[M+H]^{+}$.

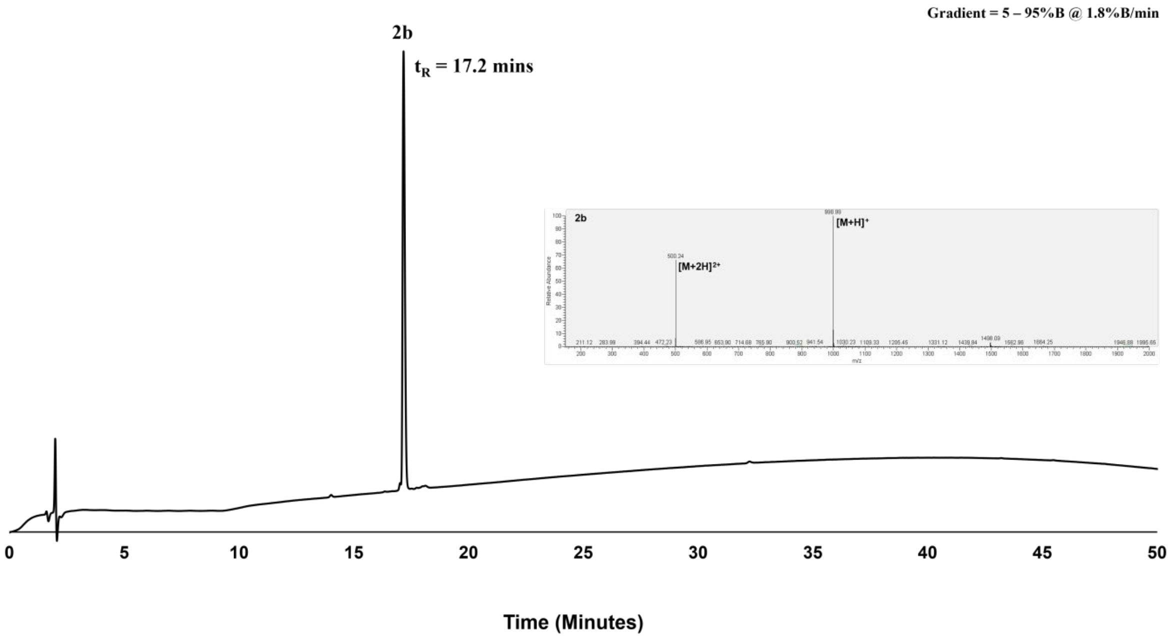

Supporting Information Figure S14. Analytical RP-HPLC chromatogram (214 nm) of purified peptide, **2b**, *ca.* 98% as analysed by peak area. **RP-HPLC:** linear gradient = 5% – 95%B over 60 min, flow rate = 1.0 mL/min, l = 214 nm; t_R = 17.2 mins. **LCMS (ESI+)** inset of **2b**, mass calculated for [$C_{43}H_{67}N_{17}O_{11}$ + H] 998.12; deconvoluted mass observed: 998.24 ± 0.35. Charge states; 500.24 $[M+2H]^{2+}$, 998.99 $[M+H]^{+}$.

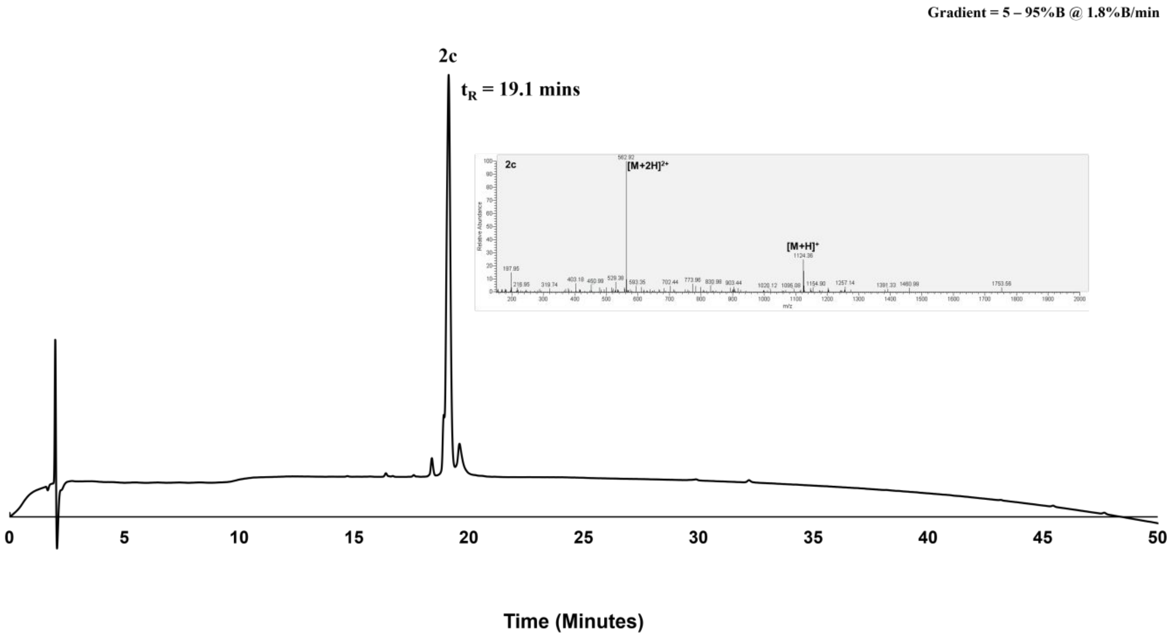

Supporting Information Figure S15. Analytical RP-HPLC chromatogram (214 nm) of purified peptide, **2c**, *ca.* 92% as analysed by peak area. **RP-HPLC:** linear gradient = 5% – 95%B over 60 min, flow rate = 1.0 mL/min, l = 214 nm; t_R = 19.1 mins. **LCMS (ESI+)** inset of **2c**, mass calculated for [$C_{43}H_{66}IN_{17}O_{11}$ + H] 1124.01; deconvoluted mass observed: 1123.60 ± 0.34. Charge states; 562.92 $[M+2H]^{2+}$, 1124.36 $[M+H]^{+}$.

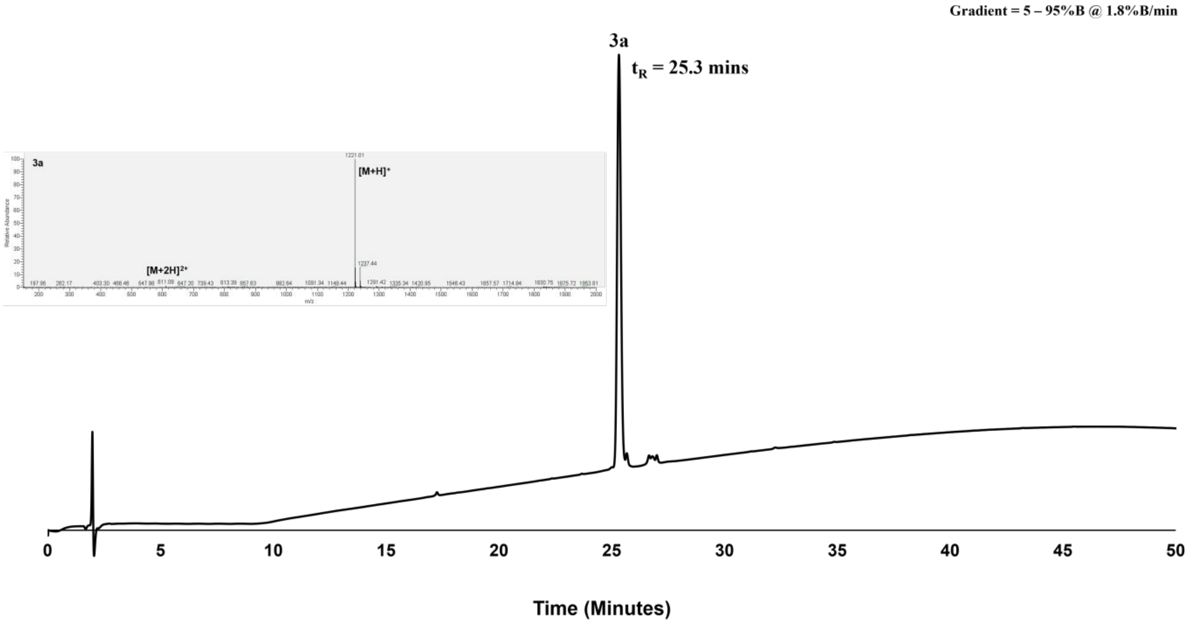

Supporting Information Figure S16. Analytical RP-HPLC chromatogram (214 nm) of purified peptide, **3a**, *ca.* 95% as analysed by peak area. **RP-HPLC:** linear gradient = 5% – 95%B over 60 min, flow rate = 1.0 mL/min, l = 214 nm; t_R = 25.3 mins. **LCMS (ESI+)** inset of **3a**, mass calculated for [$C_{55}H_{75}N_{15}O_{17}$ + H] 1220.31; deconvoluted mass observed: 1220.09 ± 0.11. Charge states; 611.08 $[M+2H]^{2+}$, 1221.01 $[M+H]^{+}$.

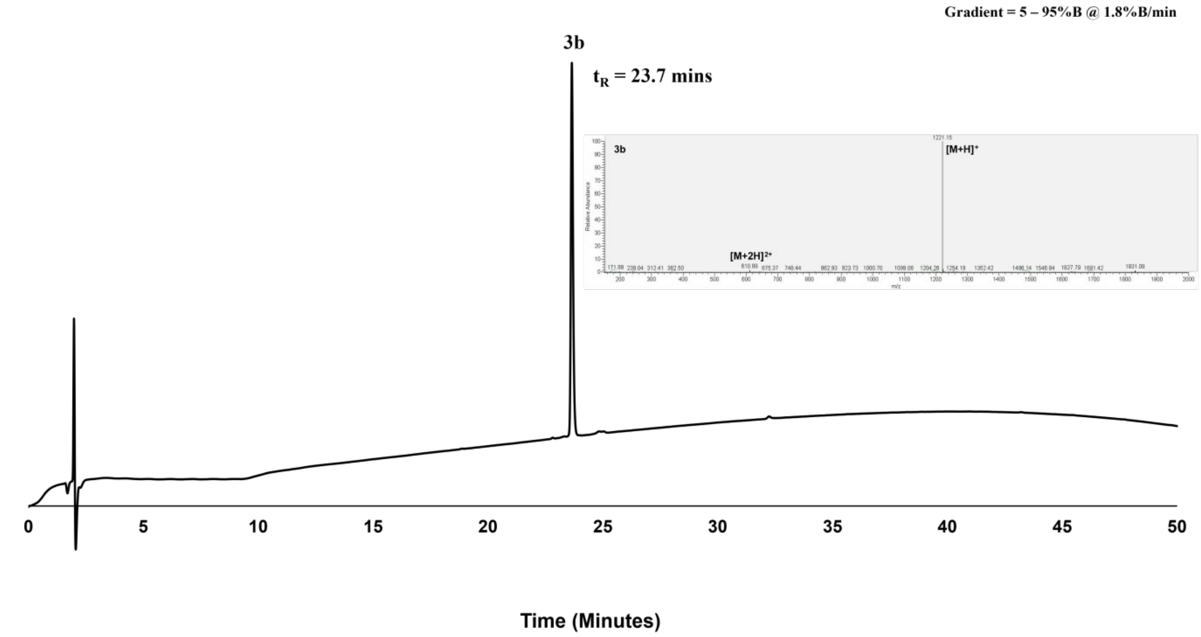

Supporting Information Figure S17. Analytical RP-HPLC chromatogram (214 nm) of purified peptide, **3b**, *ca.* 98% as analysed by peak area. **RP-HPLC:** linear gradient = 5% – 95%B over 60 min, flow rate = 1.0 mL/min, l = 214 nm; t_R = 23.7 mins. **LCMS (ESI+)** inset of **3b**, mass calculated for [$C_{55}H_{75}N_{15}O_{17}$ + H] 1220.31; deconvoluted mass observed: 1219.97 ± 0.26. Charge states; 610.89 $[M+2H]^{2+}$, 1221.15 $[M+H]^{+}$.

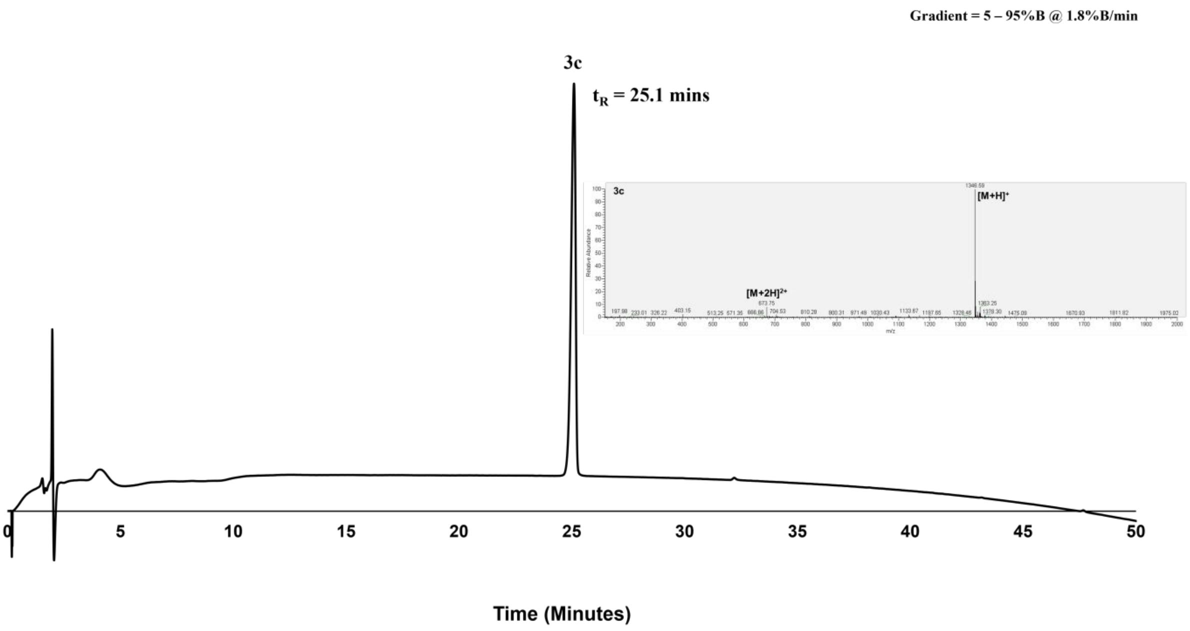

Supporting Information Figure S18. Analytical RP-HPLC chromatogram (214 nm) of purified peptide, **3c**, *ca.* 99% as analysed by peak area. **RP-HPLC:** linear gradient = 5% – 95%B over 60 min, flow rate = 1.0 mL/min, l = 214 nm; t_R = 25.1 mins. **LCMS (ESI+)** inset of **3c**, mass calculated for [$C_{55}H_{74}IN_{15}O_{17}$ + H] 1346.21; deconvoluted mass observed: 1345.55 ± 0.06. Charge states; 673.75 $[M+2H]^{2+}$, 1346.59 $[M+H]^{+}$.

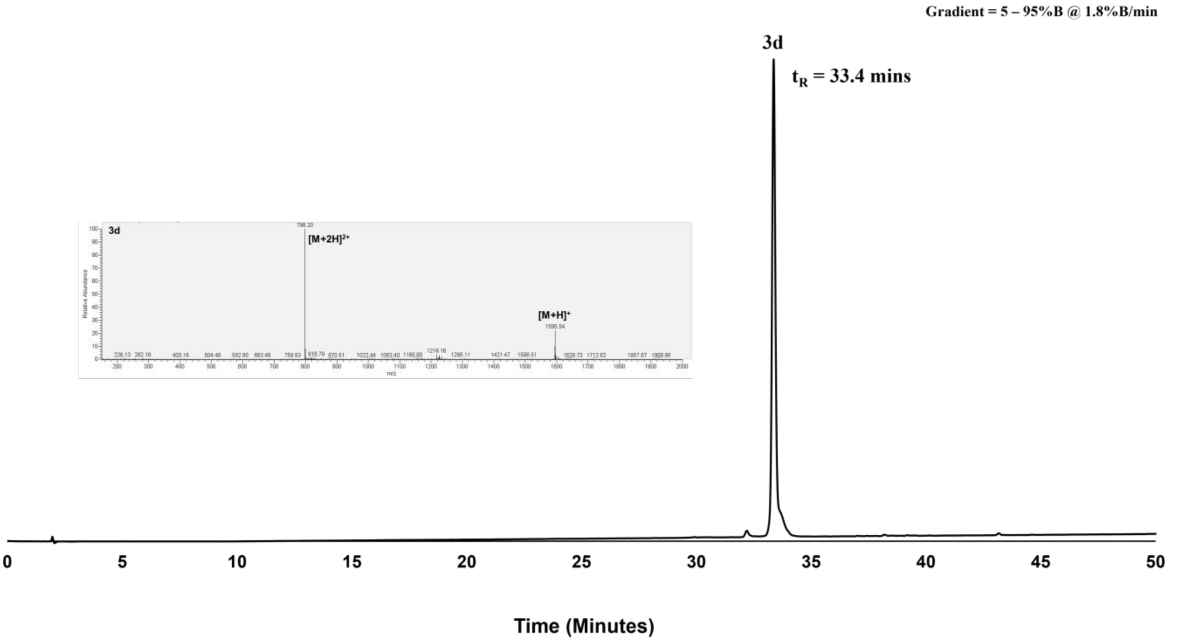

Supporting Information Figure S19. Analytical RP-HPLC chromatogram (214 nm) of purified peptide, **3d**, *ca.* 96% as analysed by peak area. **RP-HPLC:** linear gradient = 5% – 95%B over 60 min, flow rate = 1.0 mL/min, l = 214 nm; t_R = 33.4 mins. **LCMS (ESI+)** inset of **3d**, mass calculated for $C_{78}H_{95}N_{15}O_{22}$ + H] 1594.70; deconvoluted mass observed: 1594.47 ± 0.10. Charge states; 798.20 $[M+2H]^{2+}$, 1595.54 $[M+H]^{+}$.

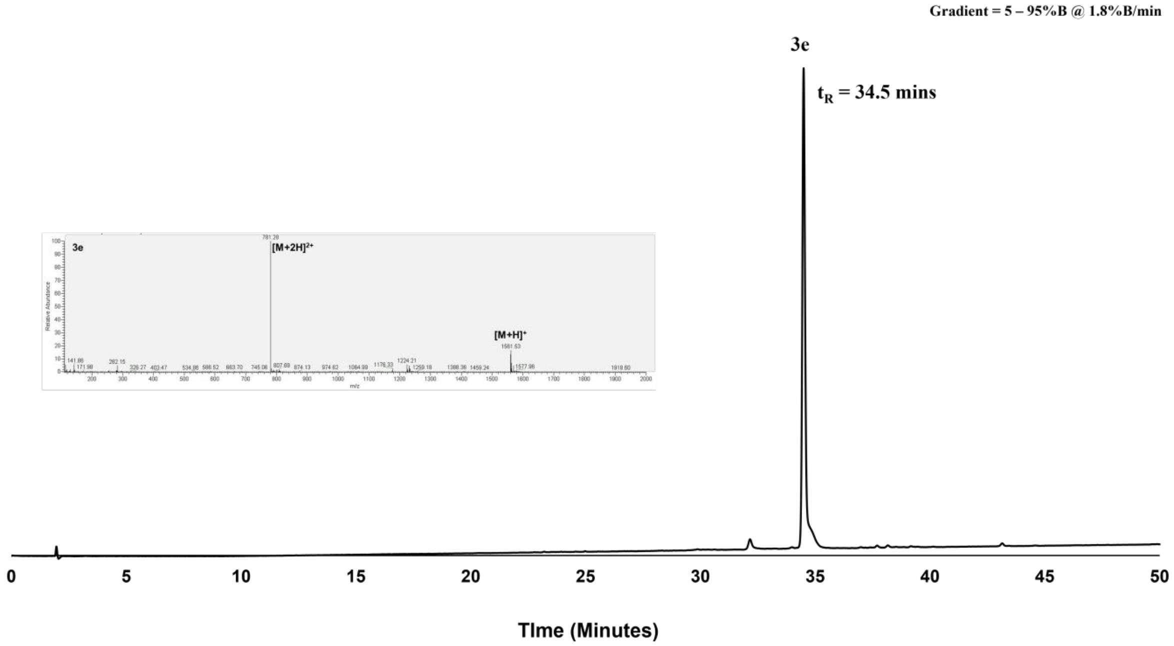

Supporting Information Figure S20. Analytical RP-HPLC chromatogram (214 nm) of purified peptide, **3e**, *ca.* 95% as analysed by peak area. **RP-HPLC:** linear gradient = 5% – 95%B over 60 min, flow rate = 1.0 mL/min, l = 214 nm; t_R = 34.5 mins. **LCMS (ESI+)** inset of **3e**, mass calculated for [$C_{78}H_{93}N_{15}O_{20}$ + H] 1560.69; deconvoluted mass observed: 1560.55 ± 0.02. Charge states; 781.28 $[M+2H]^{2+}$, 1561.53 $[M+H]^{+}$.

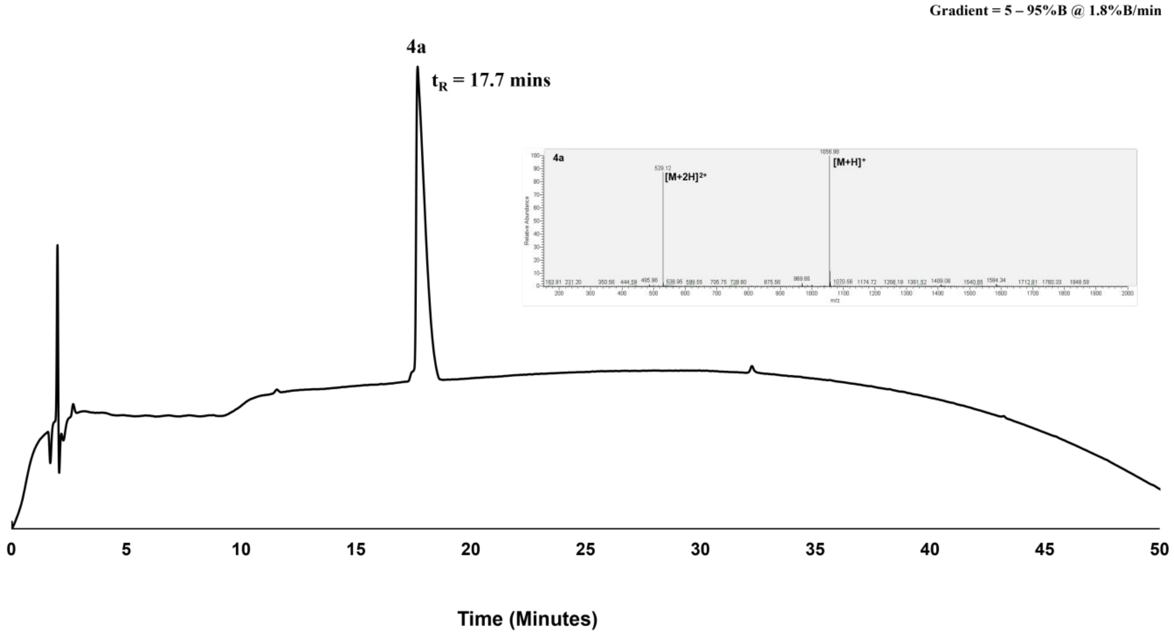

Supporting Information Figure S21. Analytical RP-HPLC chromatogram (214 nm) of purified peptide, **4a**, *ca.* 99% as analysed by peak area. **RP-HPLC:** linear gradient = 5% – 95%B over 60 min, flow rate = 1.0 mL/min, l = 214 nm; t_R = 17.7 mins. **LCMS (ESI+)** inset of **4a**, mass calculated for [$C_{44}H_{65}N_{17}O_{14}$ + H] 1056.11; deconvoluted mass observed: 1056.11 ± 0.18. Charge states; 529.12 $[M+2H]^{2+}$, 1056.98 $[M+H]^{+}$.

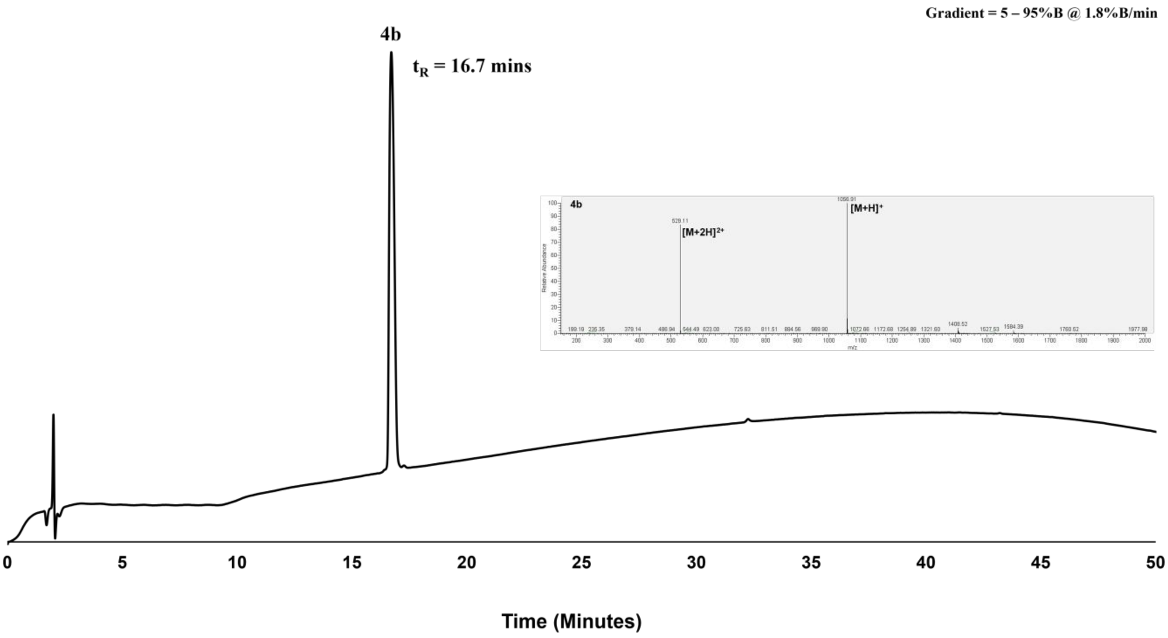

Supporting Information Figure S22. Analytical RP-HPLC chromatogram (214 nm) of purified peptide, **4b**, *ca.* 99% as analysed by peak area. **RP-HPLC:** linear gradient = 5% – 95%B over 60 min, flow rate = 1.0 mL/min, l = 214 nm; t_R = 16.7 mins. **LCMS (ESI+)** inset of **4b**, mass calculated for [$C_{44}H_{65}N_{17}O_{14}$ + H] 1056.11; deconvoluted mass observed: 1056.07 ± 0.22. Charge states; 529.11 $[M+2H]^{2+}$, 1056.91 $[M+H]^{+}$.

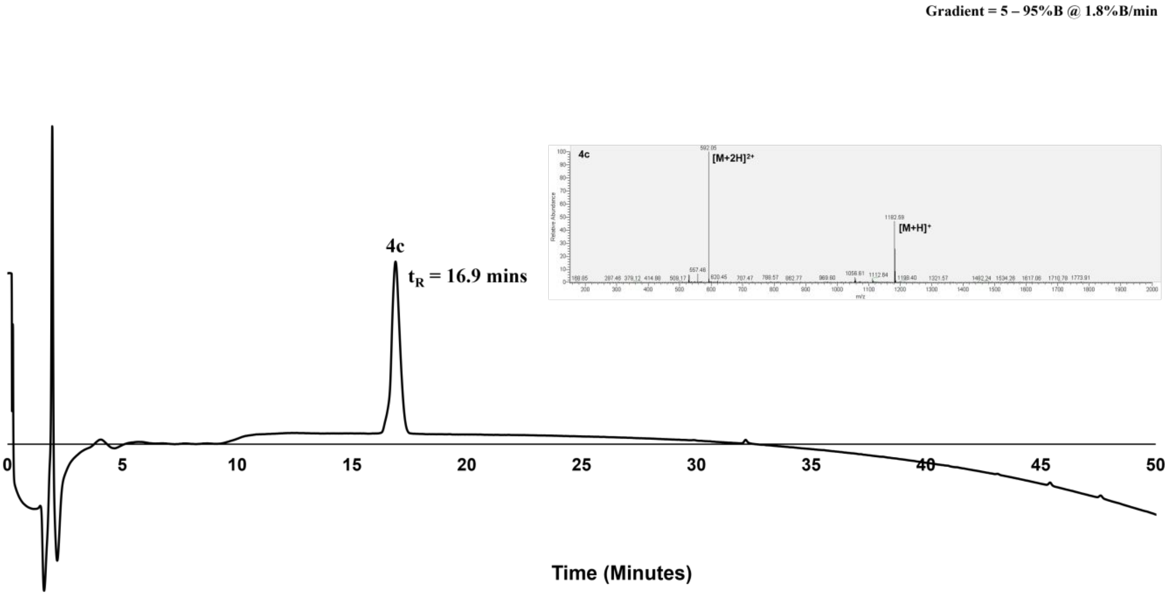

Supporting Information Figure S23. Analytical RP-HPLC chromatogram (214 nm) of purified peptide, **4c**, *ca.* 98% as analysed by peak area. **RP-HPLC:** linear gradient = 5% – 95%B over 60 min, flow rate = 1.0 mL/min, l = 214 nm; t_R = 17.0 mins. **LCMS (ESI+)** inset of **4c**, mass calculated for [$C_{44}H_{64}IN_{17}O_{14}$ + H] 1182.01; deconvoluted mass observed: 1181.85 ± 0.36. Charge states; 592.05 $[M+2H]^{2+}$, 1182.59 $[M+H]^{+}$.

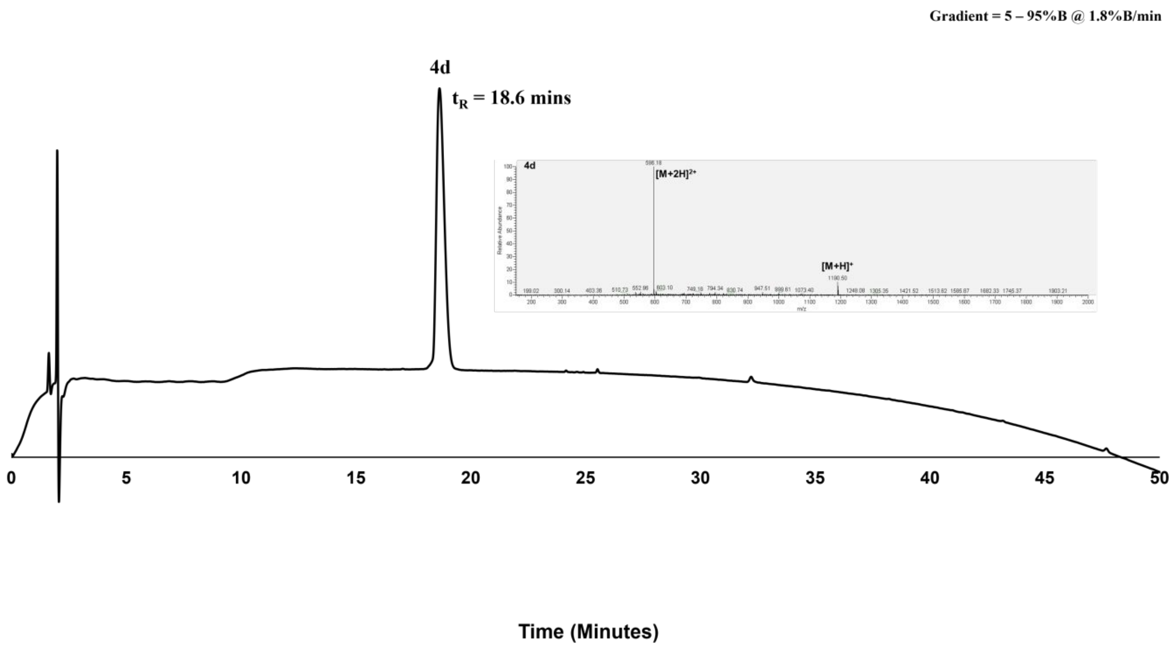

Supporting Information Figure S24. Analytical RP-HPLC chromatogram (214 nm) of purified peptide, **4d**, *ca.* 99%) as analysed by peak area. **RP-HPLC:** linear gradient = 5% – 95%B over 60 min, flow rate = 1.0 mL/min, l = 214 nm; t_R = 18.6 mins. **LCMS (ESI+)** inset of **4d**, mass calculated for [$C_{52}H_{71}N_{17}O_{16}$ + H] 1190.24; deconvoluted mass observed: 1189.91 ± 0.64. Charge states; 596.18 $[M+2H]^{2+}$, 1190.50 $[M+H]^{+}$.

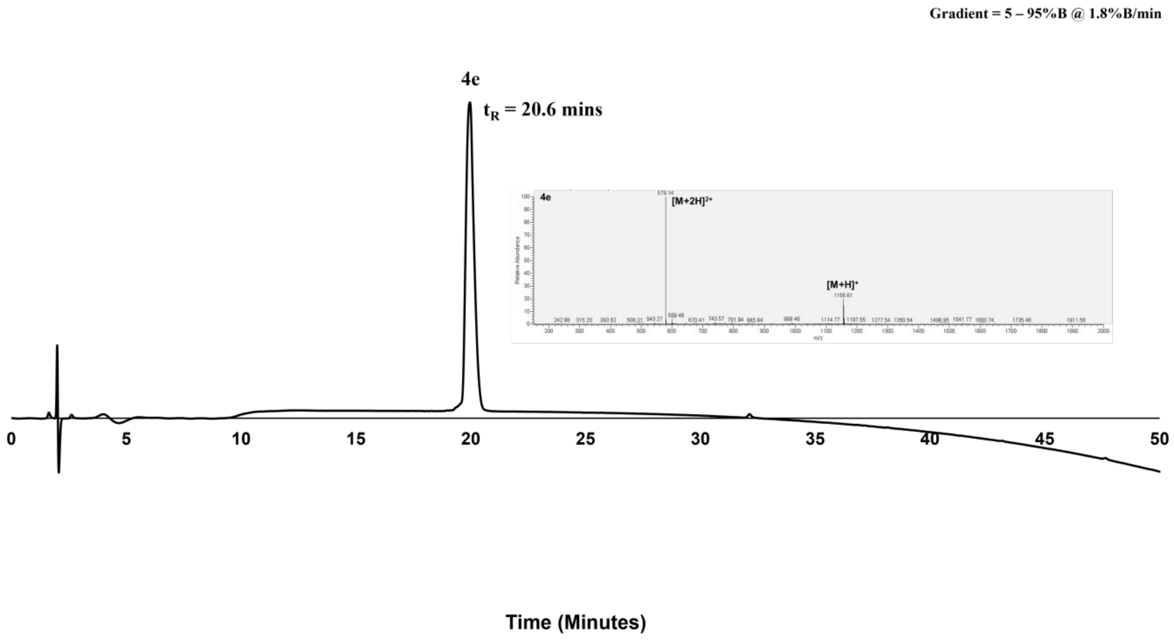

Supporting Information Figure S25. Analytical RP-HPLC chromatogram (214 nm) of purified peptide, **4e**, *ca.* 98% as analysed by peak area. **RP-HPLC:** linear gradient = 5% – 95%B over 60 min, flow rate = 1.0 mL/min, l = 214 nm; t_R = 20.6 mins. **LCMS (ESI+)** inset of **4e**, mass calculated for [$C_{52}H_{69}N_{17}O_{14}$ + H] 1156.23; deconvoluted mass observed: 1155.95 ± 0.47. Charge states; 579.14 $[M+2H]^{2+}$, 1156.61 $[M+H]^{+}$.

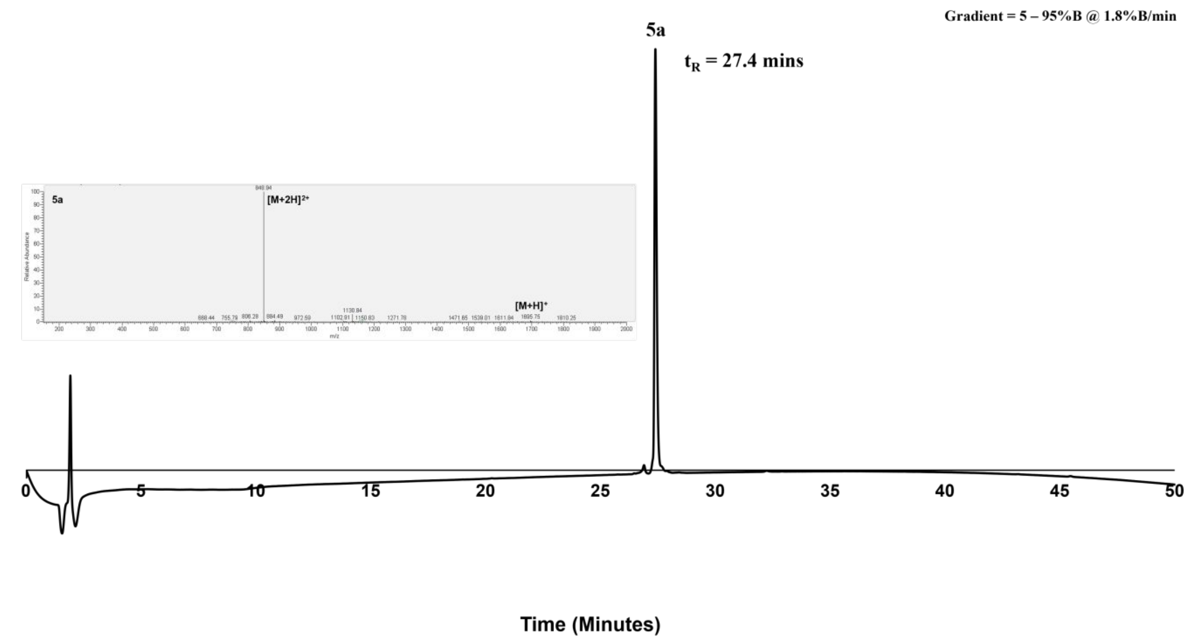

Supporting Information Figure S26. Analytical RP-HPLC chromatogram (214 nm) of purified peptide, **5a**, *ca.* 98% as analysed by peak area. **RP-HPLC:** linear gradient = 5% – 95%B over 60 min, flow rate = 1.0 mL/min, l = 214 nm; t_R = 27.4 mins. **LCMS (ESI+)** inset of **5a**, mass calculated for [$C_{81}H_{110}N_{22}O_{19}$ + H] 1695.91; deconvoluted mass observed: 1695.32 ± 0.80. Charge states; 848.94 $[M+2H]^{2+}$, 1695.75 $[M+H]^{+}$.

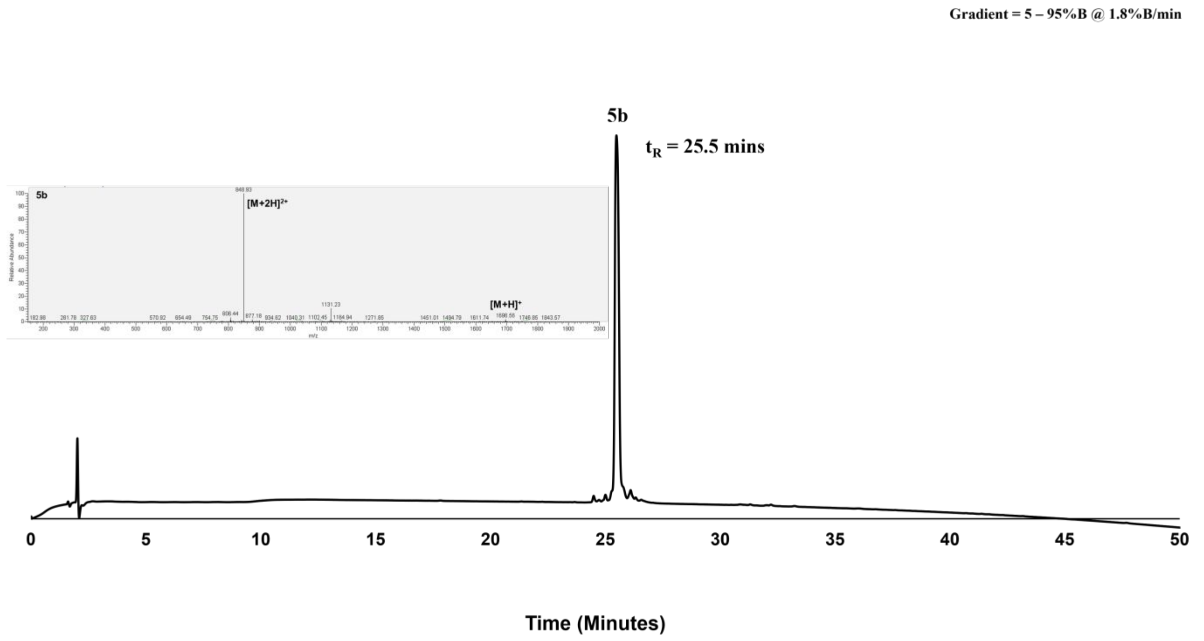

Supporting Information Figure S27. Analytical RP-HPLC chromatogram (214 nm) of purified peptide, **5b**, *ca.* 98% as analysed by peak area. **RP-HPLC:** linear gradient = 5% – 95%B over 60 min, flow rate = 1.0 mL/min, l = 214 nm; t_R = 25.5 mins. **LCMS (ESI+)** inset of **5b**, mass calculated for [$C_{81}H_{110}N_{22}O_{19}$ + H] 1695.91; deconvoluted mass observed: 1695.71 ± 0.21. Charge states; 848.93 $[M+2H]^{2+}$, 1696.56 $[M+H]^{+}$.

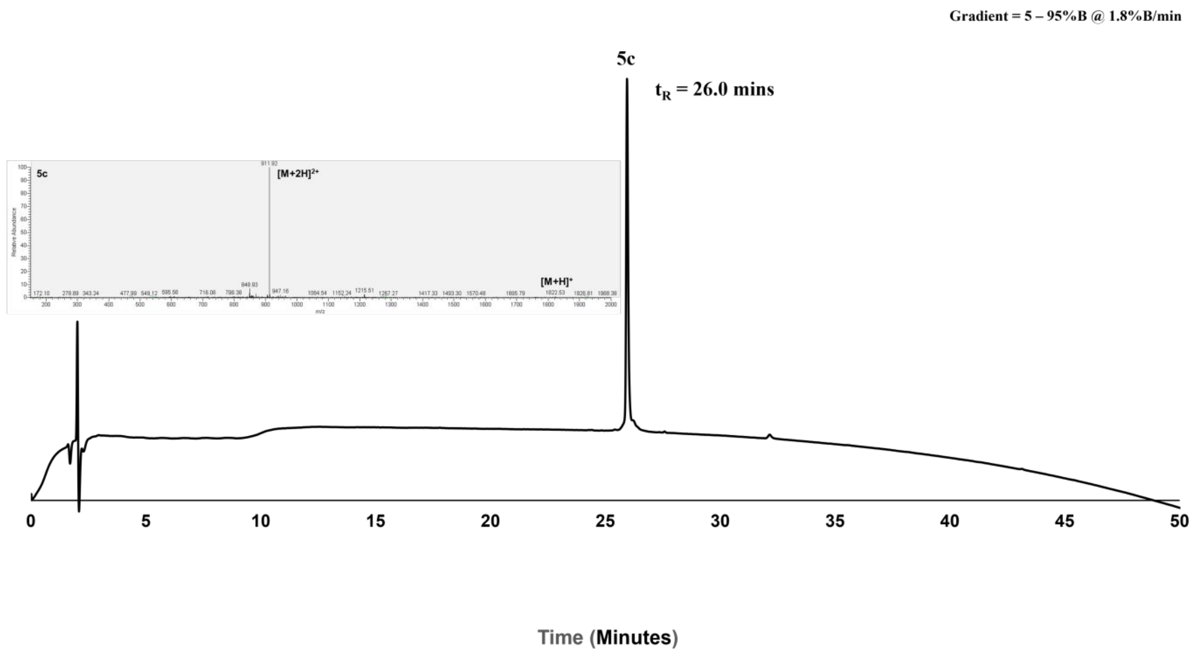

Supporting Information Figure S28. Analytical RP-HPLC chromatogram (214 nm) of purified peptide, **5c**, *ca.* 95% as analysed by peak area. **RP-HPLC:** linear gradient = 5% – 95%B over 60 min, flow rate = 1.0 mL/min, l = 214 nm; t_R = 26.0 mins. **LCMS (ESI+)** inset of **5c**, mass calculated for [$C_{81}H_{109}IN_{22}O_{19}$ + H] 1821.80; deconvoluted mass observed: 1821.69 ± 0.22. Charge states; 911.92 $[M+2H]^{2+}$, 1822.53 $[M+H]^{+}$.

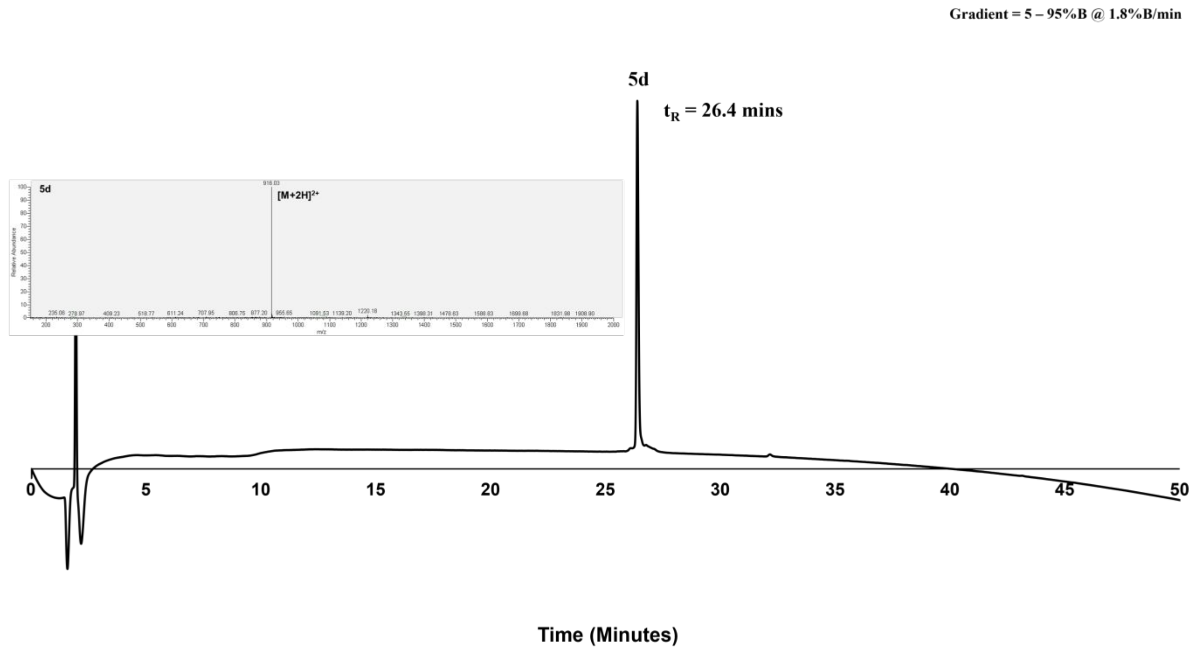

Supporting Information Figure S29. Analytical RP-HPLC chromatogram (214 nm) of purified peptide, **5d**, *ca.* 96% as analysed by peak area. **RP-HPLC:** linear gradient = 5% – 95%B over 60 min, flow rate = 1.0 mL/min, l = 214 nm; t_R = 26.4 mins. **LCMS (ESI+)** inset of **5d**, mass calculated for [$C_{89}H_{116}N_{22}O_{21}$ + H] 1830.04; deconvoluted mass observed: 1830.06. Charge states; 918.03 $[M+2H]^{2+}$.

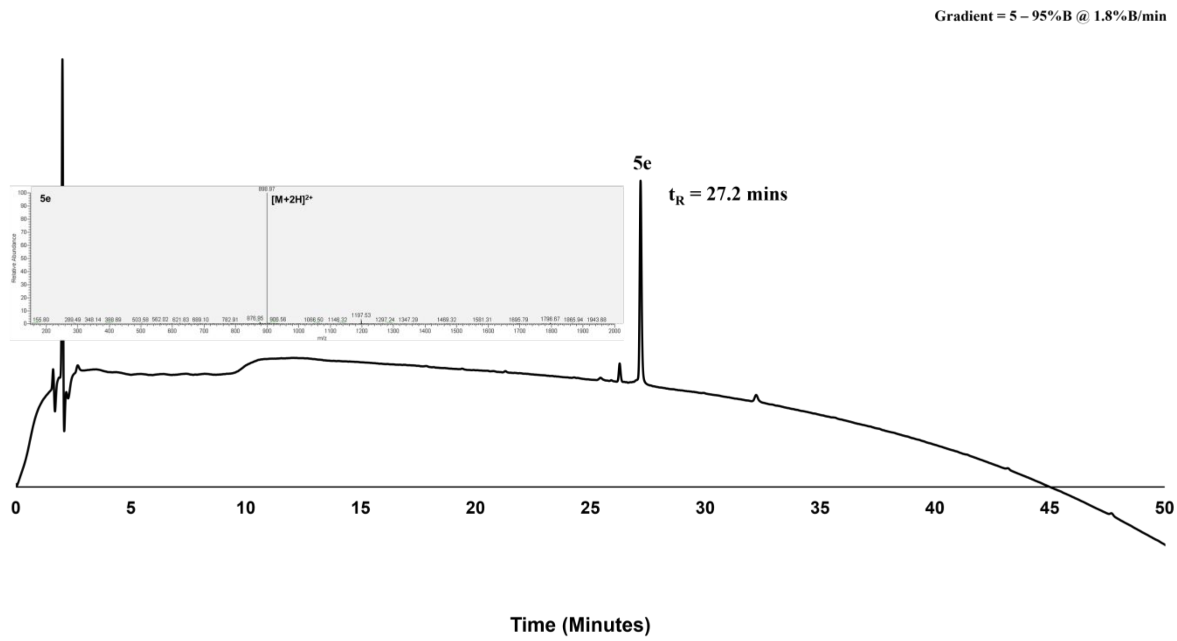

Supporting Information Figure S30. Analytical RP-HPLC chromatogram (214 nm) of purified peptide, **5e**, *ca.* 95% as analysed by peak area. **RP-HPLC:** linear gradient = 5% – 95%B over 60 min, flow rate = 1.0 mL/min, l = 214 nm; t_R = 27.2 mins. **LCMS (ESI+)** inset of **5e**, mass calculated for [$C_{89}H_{114}N_{22}O_{19}$ + H] 1796.03; deconvoluted mass observed: 1795.94. Charge states; 898.97 $[M+2H]^{2+}$.

References

(1) Shepperson, O. A.; Malone, M. A.; Arnott, K. I. M.; Brown, R. J.; Jamieson, A. G. On-Resin Synthesis and Late-Stage Functionalization of Macrocyclic Atosiban Mimetics via 5-Iodo-1,4-Triazoles. *Org. Lett.* **2025**, *27* (40), 11249–11253. https://doi.org/10.1021/acs.orglett.5c03507.
